# An integrative coding and non-coding SNPs analysis of the *CLDN-3* gene in humans to identify high-priority variants using *in-silico* analysis

**DOI:** 10.3389/fonc.2026.1816524

**Published:** 2026-06-29

**Authors:** Pooja Singh, Pallavi Somvanshi

**Affiliations:** School of Computational & Integrative Sciences (SCIS), Jawaharlal Nehru University, New Delhi, India

**Keywords:** CLDN-3, tight junction protein, coding and non-coding SNPs, high-priority variant, gynaecological cancers, *in-silico*

## Abstract

**Background:**

By creating intramembrane tight-junction barriers, claudin-3 (*CLDN-3*), a vital member of the claudin protein family, helps to preserve cell polarity. Through aberrant signaling pathways, the dysregulation of *CLDN-3* has been linked to gynecological and other epithelial cancers. While previous studies have explored its role in cancer progression, the functional impact of both coding and non-coding single-nucleotide polymorphisms (SNPs) in *CLDN-3* remains largely unexplored.

**Methods:**

In this study, *in-silico* tools were employed to identify high-priority variants for gynecological and other epithelial cancers.

**Results and discussion:**

Using SIFT, PolyPhen-2, and SNPs&GO, we first assessed 140 non-synonymous SNPs (nsSNPs). Out of these, 29 were consistently predicted as high-priority pathogenic by all three tools. Stability analysis based on ΔΔG using I-Mutant 2.0 and MUpro identified six corensSNPs that were stabilized after mutation and were predicted to impair protein function by MutPred2. Among them, N140I and Y147C lie near the *CLDN-3*/clostridium perfringens enterotoxin (CPE) binding domain, suggesting a novel mechanism that may interfere with CPE-based therapeutics. These predictions were further supported by molecular dynamics (MD) simulation, which shows that these mutations may compromise *CLDN-3* structural integrity and function. For non-coding SNPs analysis, we prioritized 28 high-impact untranslated region (UTR) variants out of 256 based on RegulomeDB scores (≤ 2b). Among these, four SNPs in the 3′ UTR were found to influence miRNA binding, indicating dual targetgain/loss effects that could affect post-transcriptional regulation. GTEx-based eQTL analysis identified three 5′ UTR SNPs that significantly alter *CLDN-3* expression levels, indicating high-value effects on transcriptional regulation. We used the TRANSFAC database to perform transcription factor binding site (TFBS) analysis in order to further evaluate the regulatory landscape; no noteworthy binding motifs were found. Further, minimum free energy (MFE) analysis revealed five SNPs that significantly alter mRNA secondary structure and stability, providing additional insights into the post-transcriptional regulation of *CLDN-3*.

**Conclusion:**

These comprehensively identified *in silico* coding and non-coding SNPs may serve as highpriority biomarkers requiring further experimental validation for future clinical applications.

## Graphical Abstract

**Figure:**
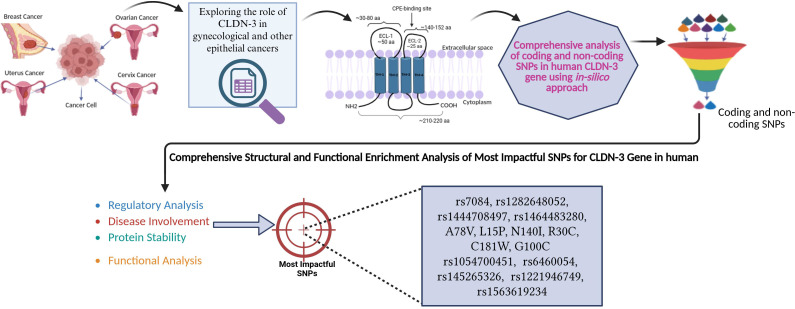

## Introduction

The most frequent form of genetic variation in humans is single-nucleotide polymorphism (SNP) variant, which ranges from 2 to 5 million (1). SNP variants are a type of polymorphism in which a single nucleotide varies between individuals. SNPs in coding regions change amino acid sequences, leading to alterations in protein function via structural changes, and are called non-synonymous SNPs (nsSNPs) (1–4). Whereas SNP variants in highly structured non-coding regions can affect transcription factor binding site (TFBS) in the 5’ untranslated region (UTR) and alter microRNA (miRNA) binding in the 3’ UTR, leading to changes in gene expression that contribute to disease progression (5).

Claudin-3 (*CLDN-3*) belongs to the family of claudin proteins and is a key component of transmembrane tight junction (TJ) proteins (6). This protein is crucial in cell-cell junction formation, which maintains cell polarity by forming an intramembrane blockade (6, 7). TJ proteins are essential for maintaining the epithelial barrier and protecting our cells, organs, and body systems from invaders (7). Notably, numerous studies have found that dysregulation of TJ protein *CLDN-3* is associated with the progression of various gynaecological cancers, including breast, ovarian, cervical, and endometrial cancers, as well as numerous other types of epithelial cancer (8–11). Therefore, targeting *CLDN-3* for drug therapy is important. Additionally, it is essential to consider its SNPs, as single-nucleotide changes in coding and non-coding regions can alter regulatory elements and thereby affect gene expression. *CLDN-3*, located on chromosome 7, specifically at the cytogenic band q11.23, has four transmembrane domains, two extracellular loops, where the C-terminal domain of Clostridium perfringens enterotoxin (CPE) interacts, and both C-terminal and N-terminal domains (7, 12). Experimental evidence has shown that the C-terminal of CLDN-3 interacts with the N-terminal of ZO-1 in its PDZ1 domain, forming a key component of TJ complexes (13). *ZO-1* also interacts with actin and provides a platform for interactions with other TJ proteins, such as *ZO-2/3*, which are essential for TJ formation (13, 14). Other studies have demonstrated that dysregulation of *CLDN-3/4* leads to the loss of E-cadherin function (15). E-cadherin, an adherens junction protein, binds to *β-catenin* and *ZO-1* at the plasma membrane, inhibiting signalling pathways initiated by β-catenin, such as Wnt and Akt. Loss of E-cadherin triggers the dissociation of β-catenin, leading to its accumulation in the cytoplasm. This accumulation enables β-catenin to enter the nucleus, where it activates transcription factors (TFs) such as *SNAIL*, *SLUG*, and *TCF*. These TFs, in turn, activate signalling pathways that drive cell proliferation and epithelial-to-mesenchymal transition (EMT), which are key processes underlying the development of epithelial neoplasms (15, 16). Any mutation in *CLDN-3* that weakens or strongly strengthens the binding between its adjacent TJ proteins should be explored, as it might disrupt homogeneous cell-cell TJ formation and compromise the integrity of the cell junction.

An *in-silico* study has been conducted to identify all possible SNPs that lead to structural and functional changes in the *CLDN-3* protein. With its C-terminal domain, CPE binds with claudins, including *CLDN-3, which* has been explored for claudin-targeted cancer therapy (12). So, SNPs that align near the CPE binding region could represent high-priority therapeutic targets for gynaecological or epithelial cancers. Through our study, we may identify and predict high-priority SNPs that could serve as therapeutic targets in gynaecological or epithelial cancers after experimental validation. In this study, we shortlisted the most significant coding and non-coding SNPs and conducted downstream analyses to assess their impact on the structure and function of the *CLDN-3* protein. To identify the most deleterious, pathogenic, and disease-causing coding SNPs, three tools were employed: Sorting Intolerant From Tolerant (17) (SIFT), Polymorphism Phenotyping v2 (18) (PolyPhen-2), and SNPs & Go (19). The SIFT prediction method assumes that functionally important amino acids are conserved across homologous sequences. Any physico-chemical change at these conserved positions is more likely to be deleterious. SIFT scores range from 0 to 1, where mutations with scores less than 0.05 are predicted to be deleterious. The PolyPhen-2 tool uses a Naïve Bayes machine-learning model to classify mutations as pathogenic or non-pathogenic. It integrates information from three main contexts: (1) Sequence conservation, (2) Structural context, and (3) Functional annotation. Sequence conservation is assessed through multiple sequence alignment to determine evolutionary conservation of the residue. The structural context perspective examines mutation sites in terms of solvent accessibility, secondary structure, and transmembrane regions, which may affect protein stability or function. Whereas functional annotation considers the biological role of the residue, whether it is part of a phosphorylation site, ligand-binding site, or other critical functional domain. PolyPhen-2 scores also range from 0 to 1. Variants with scores below 0.5 are generally predicted as benign, whereas values closer to 1.0 indicate a higher likelihood of being deleterious. The SNPs&GO tool uses a support vector machine (SVM) approach and incorporates gene ontology (GO) annotations, along with sequence conservation and structural context. This integration makes SNPs&Go more function-oriented, allowing it to better distinguish between disease-related and non-disease SNPs. For predictive purposes, this tool uses both the probability score and the reliability index. The higher the reliability scores, the greater the likelihood that a mutation is disease-causing. Additionally, other prediction tools such as PhD-SNP (https://snps.biofold.org/phd-snp/phd-snp.html), PROVEAN (20), PANTHER (21), REVEL (22), and Rhapsody (22) have also been reported for identifying disease-causing SNPs. However, due to certain limitations, these tools were excluded from the present analysis. After identifying high-value deleterious, pathogenic, and disease-associated SNPs, a subset of “core-nsSNPs” was selected for in-depth analysis to investigate their underlying pathogenic mechanisms and structural and physicochemical properties, to assess functional alterations at the atomic level. To further support the structural stability of these core-nsSNPs, molecular dynamics (MD) simulations were performed, enabling trajectory analysis of SNP-induced variations over time. Whereas, for the non-coding SNPs, we first performed annotation to identify the most impactful variants. Subsequently, we performed downstream analyses to evaluate their effects on regulatory regions, including microRNA (miRNA) associations, expression quantitative trait loci (eQTL) analyses, transcription factor binding site (TFBS) evaluation, and mRNA fold-stability assessment. Through this integrative analysis, the identified SNPs may serve as high-priority biomarkers for future clinical applications and warrant further experimental validation.

## Materials and methodology

### Sequential analysis

#### SNPs dataset retrieval

The SNPs dataset for our target gene, *CLDN-3*, was retrieved from dbSNP Build 156 release (23) (https://www.ncbi.nlm.nih.gov/snp/) and the Ensembl release 114 (24) (https://www.ensembl.org/) databases, corresponding to the transcript ID ENST00000395145.3. After browsing for the targeted gene in humans, a missense filter was applied to identify nsSNPs, while for non-coding SNPs, 5’ UTR and 3’ UTR filters were applied. Furthermore, the protein sequences of the target gene were downloaded in FASTA format from the UniProt (25) (https://www.uniprot.org/) database using the UniProt ID O15551 for the human *CLDN-3* protein.

#### Detection of deleterious, damaging, and disease-causing nsSNPs

To predict deleterious, damaging, and disease-causing nsSNPs of the targeted gene, three tools: SIFT (17), PolyPhen-2 (18), and SNPs & Go (26) were employed with their latest version on their default setting. All these tools are in silico tools used to predict nsSNPs on protein function, and each tool has its own advantages, with some providing categorical (qualitative) predictions and others providing quantitative (probability score) predictions. Categorical outputs are easy to interpret, while quantitative scores are useful for ranking or estimating confidence. Based on the sequence-based homology hypothesis, SIFT’s score ranges from 0 to 1. It predicts deleterious amino acid substitutions with a probability score below 0.05; otherwise, it is tolerated. PolyPhen-2, based on eight sequence-based and three structure-based predictive features, predicts a damaging mutation score between 0 and 1, where high scores near 1 represent a higher probability of a variant being damaging. Further, based on a support vector machine (SVM) algorithm, SNPs & GO predict disease-causing mutants based on the reliability index. The higher the reliability index, the more confident the prediction.

#### Systemic-level protein interaction analysis

To analyse the molecular-level interaction of CLDN-3, which is essential for maintaining system homeostasis and for understanding its associated pathways and molecular functions, the STRING database (27) was used to retrieve the interaction network, and the Cytoscape tool (28) was used for network visualization.

#### Protein stability prediction

nsSNPs were run to predict protein stability after identifying the consensus high-priority disease-causing nsSNPs. Focusing on consensus nsSNPs, I-Mutant 2.0 (29) and MUpro (30), both based on the SVM algorithm, were used to predict protein stability for single amino acid mutations. The ΔΔG value was calculated to quantify the free energy changes in protein stability resulting from single amino acid substitutions. A ΔΔG value less than zero (< 0 kcal/mol) means that the protein stability has decreased, while a positive value (> 0 kcal/mol) suggests an increase. We analysed ΔΔG values to understand the impact of nsSNPs on protein stability. Since both stabilizing and destabilizing SNPs can exert distinct biological effects, we prioritized stabilizing variants based on the hypothesis that they may have a more sustained impact on disease outcomes. By selecting these stabilised mutants, referred to as core-nsSNPs, we gained insights into how nsSNPs influence protein stability, thereby facilitating the identification of high-value, clinically relevant variants and improving our understanding of disease susceptibility.

#### Prediction of the molecular mechanism underlying pathogenicity

The core-nsSNPs identified through protein stability analysis were further processed to predict their molecular mechanism of pathogenicity using the MutPred2 server (31). The MutPred2 server, based on machine learning, predicts the pathogenicity of substitutions and uses multiple neural networks to generate a final score ranging from 0 to 1. A p-value threshold of less than 0.05 was applied to identify relevant pathogenic substitutions.

### Structural analysis

#### Homology modelling

The target *CLDN-3* protein structure was unavailable on the PDB, so we used the SWISS-MODEL server (32) for homology modelling. This involved using a query sequence to identify closely related templates, aligning them, and validating the model using QMEAN and GMQE scores. QMEAN (Qualitative Model Energy Analysis) and GMQE (Global Model Quality Estimate) are scoring functions that range from 0 to 1, where higher scores indicate better model quality and greater compatibility with known structural features. In the modelling process, we selected the template with the highest sequence identity and coverage. Additionally, we generated structures for the wild-type protein along with six core-nsSNPs with high conservation scores. Further, the AlphaFold2-Colab server was also used to model the structure for the cross-check validation (33).

#### Model validation

All generated homology-modelling-based structures were validated using the PROCHECK tool (34), which assesses the stereochemical quality of a protein structure by generating a Ramachandran plot.

#### Assessment of RMSD and TM-alignment values

The RMSD values for six mutant residues after superimposition with the native protein structure were calculated using the PyMOL tool, version 3.1 (35) (https://www.pymol.org/). Further, the TM-align score was calculated using the TM-align server (36) (https://zhanggroup.org/TM-align/), which compares structural dissimilarity between the native and mutant type structures. TM-score has a value in the range 0 to 1, where a score of 1 means no dissimilarity, a score of< 0.2 means unrelated structures, and a score of > 0.5 means the same fold.

#### Chemical property analysis

The mutant residue structures were further analysed using the BIOVIA Discovery Studio Visualizer, a structural analysis tool available for download from the website (https://discover.3ds.com/discovery-studio-visualizer-download). This tool helps visualize protein structures and analyse specific core-nsSNPs based on factors such as polar and non-polar bonds, residue solvent accessibility, and the secondary structure involved.

#### Docking analysis

Since the 3-D structure of protein complexes is generally more difficult to determine experimentally, protein-protein docking is an in-silico technique that predicts the structure of complexes from the structures of individual proteins. In this study, the HADDOCK v2.4 (37) web server (https://rascar.science.uu.nl/) was employed, on default settings, to dock the wild-type CLDN-3 molecule, along with its six core-nsSNPs, to the ZO-1 protein in its PDZ1 domain, individually using their 3D structures. *ZO-1* interacts directly via its N-terminal PDZ1 domain (amino acids 17–26) with the C-terminal region of *CLDN-3* (amino acids 210–220), as identified in the literature (13, 16, 38). ZO-1 PDZ1 domain (2H3M) mainly consists of a six-stranded β-sandwich that is capped with one α-helix, which serves as a binding site for the intracellular tails of TJ proteins (13, 39). By interacting with these integral membrane proteins, the PDZ1 domain helps to recruit them to the TJ complex, which is essential for establishing the epithelial barrier function, maintaining cell polarity, and controlling the paracellular pathway (13, 16).

#### MD simulation analysis

To investigate the structural consequences of mutations at the atomic level in a dynamic context, an MD simulation was conducted for the identified core-nsSNPs of *CLDN-3*, including the wild-type. The MD simulations of the wild-type *CLDN-3* protein and its selected nsSNP variants were conducted for 100-nanosecond production runs using the CHARMM36 force field. The system was placed in a cubic box with a minimum 1.0 nm distance between the protein and the box edges, under periodic boundary conditions, and solvated using the SPC water model in GROMACS (40). The solvated system was neutralized by replacing water molecules with appropriate counterions (Na^+^/Cl^-^) using the *gmx genion* module of GROMACS, and where required, ions were added to achieve a physiological salt concentration. Further, the system was energy-minimized and equilibrated for 100 picoseconds under NVT and NPT ensembles at 300 K and 1 bar, respectively, prior to the production simulation. The MD simulation analysis encompassed the evaluation of root-mean-square deviation (RMSD), root-mean-square fluctuations (RMSF), radius of gyration (Rg), and intramolecular hydrogen bonding (Hb). These analyses provided insights into various aspects of protein structure, including alterations in flexibility, rigidity, and compactness. The trajectory analysis of the wild-type *CLDN-3* and its six mutants was conducted using different GROMACS tools. Specifically, RMSD, RMSF, Rg, and intramolecular hydrogen bonding were calculated using the *gmx rms*, *gmx rmsf*, *gmx gyrate*, and *gmx hbond* tools.

#### RegulomeDB analysis

After retrieving 5’ and 3’ UTR SNPs, functional annotation was performed using the RegulomeDB (41) database. RegulomeDB integrates high-throughput experimental datasets from ENCODE and other sources, along with computational predictions and manual annotations, to identify putative regulatory potential and functional variants. Variants with a RegulomeDB score of ≤ 2b were selected for further analysis, as they are likely to affect TFB and influence gene expression.

#### miRNA-SNP association analysis

To identify SNPs associated with miRNA and their effects on target gain or loss as potential biomarkers, the miRNASNP-v3 (42) database was employed. SNP IDs were used as input for the analysis, and variants exhibiting both target gain and loss properties were selected as significant SNPs.

#### eQTL assessment

To examine the distribution of gene expression levels across different genotypes at specific variants, expression quantitative trait loci (eQTL) analysis was conducted using the Genotype-Tissue Expression (GTEx) database (43). For this analysis, SNP IDs from the UTRs were used as input. Variants showing a consistent trend in gene expression across genotypes and a p-value ≤ 0.05 were considered significant SNPs.

#### TFBS evaluation

To see the impact of 5’ UTR SNPs on TFBSs, the TRANSFAC database (44) (https://genexplain.com/transfac-product/), SNP2TFBS (45) web interface from the Swiss Bioinformatics Resource Portal (expasy.org), and ChIP-Atlas 3.0 were utilized. Variants in VCF format and SNP IDs were used as inputs for the analysis.

#### RNA fold analysis

To assess the impact of SNPs on mRNA secondary structure, the RNAfold (46) web server (http://rna.tbi.univie.ac.at/cgi-bin/RNAWebSuite/RNAfold.cgi) was used. Wild-type and mutant sequences of the variants were provided as input for the analysis, and changes in minimum free energy (MFE) were used as a parameter to evaluate alterations in mRNA secondary structure.

## Results

### SNPs datasets

A total of 1,578 SNPs were identified in the *CLDN-3* gene using the dbSNP and Ensembl databases. Among these, 140 variants were nsSNPs that directly affect the amino acid sequence and therefore potentially alter protein structure and function. In addition, a substantial proportion of variants were located in regulatory regions, with 150 SNPs in the 3′ UTR and 106 SNPs in the 5′ UTR. The enrichment of SNPs in both coding and untranslated regions suggests that *CLDN-3* regulation may be influenced not only at the protein level but also at the post-transcriptional and translational levels. Such non-coding variants may affect mRNA stability, translational efficiency, or regulatory element binding, thereby indirectly modulating *CLDN-3* expression. A detailed classification and distribution of these variants are presented in **Supplementary Tables 1**, **2** and **Supplementary Figure 1**.

### Detection of deleterious, damaging, and disease-causing nsSNPs

**Figure 1:**
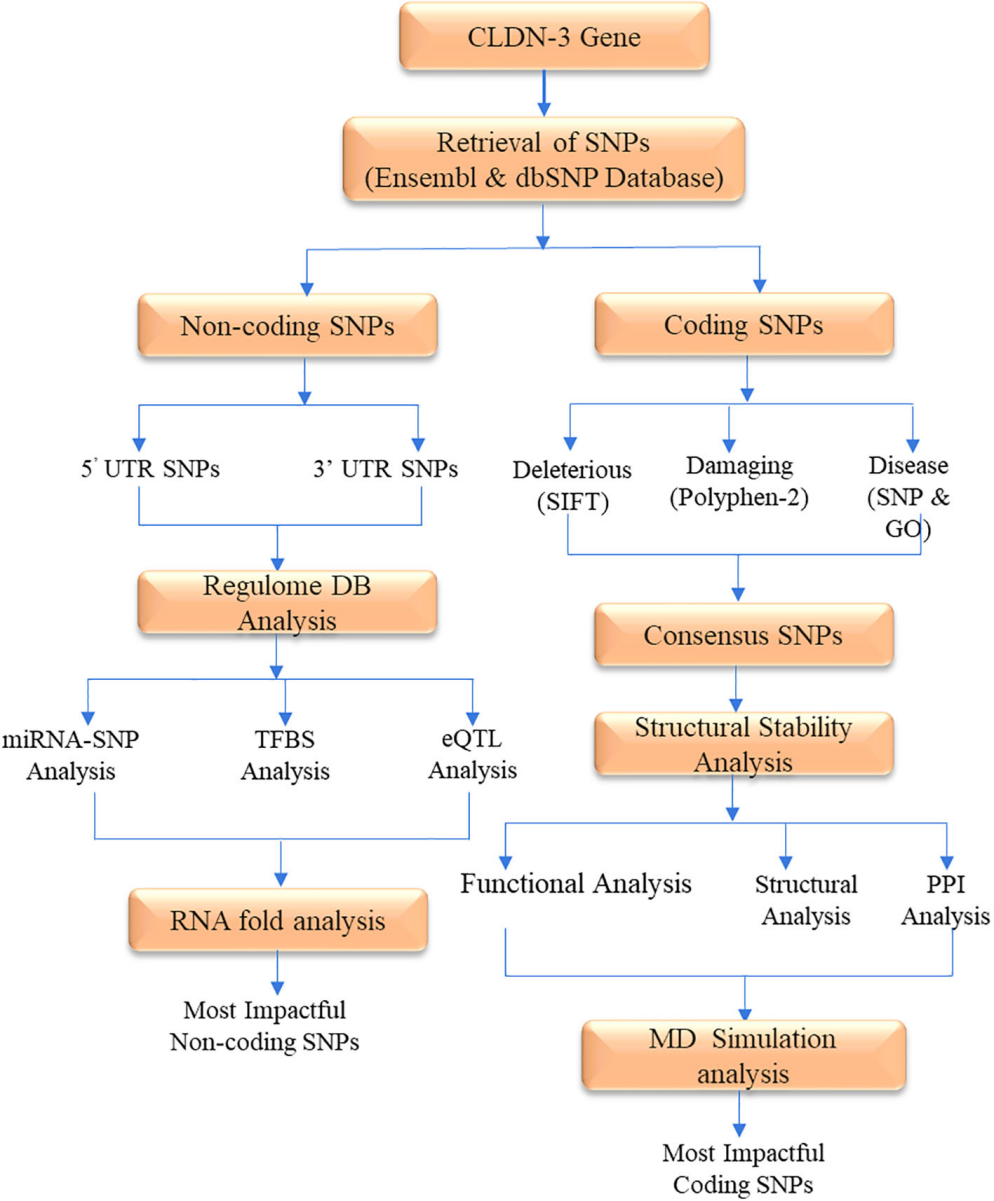
Schematic representation of the methodology employed in present study.

After running SIFT, PolyPhen-2, and SNP&GO on the missense mutations in CLDN-3, we found that 29 of 140 were consensus disease-causing nsSNPs across all three tools. For further details, please refer to **Supplementary Table 1** provides comprehensive information on the identified nsSNPs for *CLDN-3*. The consensus nsSNPs were selected as deleterious, predicted by all three tools, to ensure high confidence in variant pathogenicity. SNPs predicted to be neutral or non-deleterious by all three tools were not considered consensus for further analyses. A detailed description, including the scores and predictions for the sorted nsSNPs, is provided in **Table 1**, and the utilized pipeline is shown in **Figure 1**.

**Table 1:** 29 most deleterious functional coding SNPs out of 140 predicted by different prediction tools for CLDN-3.

S.No	nsSNP ID	Amino acid change	SIFT score	SIFT prediction	PolyPhen2 score	PolyPhen2 prediction	SNP &GO index	SNP &GO prediction
1	rs11549498	R30C	0	Deleterious	1	Damaging	4	Disease
2	rs139191328	P134Q	0	Deleterious	1	Damaging	2	Disease
3	rs141257286	I143N	0	Deleterious	0.988	Damaging	5	Disease
4	rs201650771	D75H	0.04	Deleterious	1.000	Damaging	0	Disease
5	rs781999702	L15P	0	Deleterious	0.994	Damaging	4	Disease
6	rs782211762	C24G	0.03	Deleterious	1	Damaging	7	Disease
7	rs782235875	L129R	0	Deleterious	0.565	Damaging	2	Disease
8	rs782350219	Q62R	0.02	Deleterious	0.84	Damaging	6	Disease
9	rs782368222	V107E	0.02	Deleterious	0.969	Damaging	2	Disease
10	rs782664344	V119E	0.02	Deleterious	0.635	Damaging	2	Disease
11	rs1168202234	G93R	0.01	Deleterious	0.721	Damaging	3	Disease
12	rs1199877723	A128P	0.01	Deleterious	1	Damaging	3	Disease
13	rs1243569776	A78V	0.01	Deleterious	0.590	Damaging	1	Disease
14	rs1375975812	K64E	0	Deleterious	0.750	Damaging	5	Disease
15	rs1397705319	R144W	0	Deleterious	0.998	Damaging	2	Disease
16	rs1401007590	C181W	0	Deleterious	0.689	Damaging	5	Disease
17	rs1554626603	Y219S	0	Deleterious	1	Damaging	4	Disease
18	rs1554626657	V151G	0	Deleterious	0.509	Damaging	1	Disease
19	rs1554626663	P149L	0	Deleterious	0.73	Damaging	4	Disease
20	rs1554626665	Y147C	0	Deleterious	0.999	Damaging	2	Disease
21	rs1554626675	N140I	0	Deleterious	1.0	Damaging	3	Disease
22	rs1554626735	S68L	0	Deleterious	0.581	Damaging	5	Disease
23	rs1554626749	A33T	0.01	Deleterious	0.65	Damaging	2	Disease
24	rs1554626750	P27R	0	Deleterious	0.984	Damaging	6	Disease
25	rs1554626752	P27S	0	Deleterious	1	Damaging	4	Disease
26	rs782101922	G100C	0	Deleterious	1	Damaging	5	Disease
27	rs1554626763	W17C	0	Deleterious	1	Damaging	4	Disease
28	rs781905709	G177S	0	Deleterious	1	Damaging	2	Disease
29	rs113016282	I21N	0	Deleterious	1	Damaging	1	Disease

### Systemic-level protein interaction analysis

**Figure 2:**
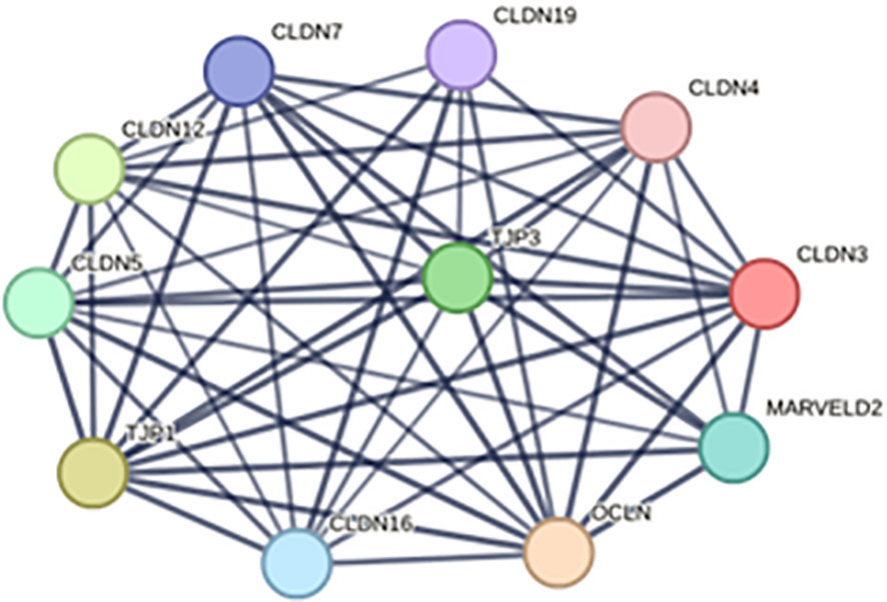
CLDN-3 protein interaction network analysis. Network representation illustrating the interactions of CLDN-3 with associated tight junction proteins, including CLDN family members, TJP proteins, OCLN, and MARVELD2, highlighting its role in maintaining epithelial barrier integrity.

Using the Cytoscape network visualization tool, it was discovered that *CLDN-3* jointly interacts with ten other TJ proteins, including *CLDN-4*, *CLDN-5*, *CLDN-7*, *CLDN-12*, *CLDN-19*, *CLDN-16*, *OCLN*, *MARVELD2*, *TJP-1*, and *TJP-3*, with a high confidence score of 0.70 and an average clustering coefficient of 0.945, as shown in **Figure 2**. These interactions are involved in pathways related to TJs, leukocyte trans-endothelial migration, and cell adhesion molecules. Consequently, they play critical roles in cell adhesion molecule binding, protein domain-specific binding, and structural molecule and protein binding activities. Any alterations in these TJ proteins could disrupt the entire network, potentially leading to the dysfunction of the associated pathways and molecular functions.

### Protein stability prediction

Twenty-nine consensus disease-causing nsSNPs were analysed using I-Mutant 2.0 and MUpro server to assess their effects on protein stability. While all SNPs indicated a decrease in protein stability, some nsSNPs that showed increased stability, A78V, C181W, N140I, G100C, Y147C, and A128P, referred to core-nsSNPs for *CLDN-3*, are shown in **Table 2**. These six core-nsSNPs with increased ΔΔG energy were assessed for further sequential and structural analysis.

**Table 2:** Protein stability prediction for CLDN-3 most deleterious nsSNPs.

S.No.	CLDN-3 (SNP-ID)	Amino acid mutation	MUpro ΔΔG	MUpro prediction	I-Mutant2.0 ΔΔG	I-Mutant2.0 prediction
1	rs11549498	R30C	-0.0807	Decrease	-0.75	Decrease
2	rs139191328	P134Q	-0.2287	Decrease	-1.89	Decrease
3	rs141257286	I143N	-1.54	Decrease	-2.48	Decrease
4	rs201650771	D75H	-0.942	Decrease	-1.01	Decrease
5	rs781999702	L15P	-1.619	Decrease	-1.20	Decrease
6	rs782211762	C24G	-1.55	Decrease	-1.10	Decrease
7	rs782235875	L129R	-2.32	Decrease	-2.17	Decrease
8	rs782350219	Q62R	-0.338	Decrease	-1.28	Decrease
9	rs782368222	V107E	-1.241	Decrease	-0.22	Decrease
10	rs782664344	V119E	-1.507	Decrease	-1.48	Decrease
11	rs1168202234	G93R	-0.484	Decrease	-1.34	Decrease
12	rs1199877723	A128P	-2.01	Decrease	1.01	**Increase**
13	rs1243569776	A78V	0.2922	**Increase**	1.06	**Increase**
14	rs1375975812	K64E	-0.0874	Decrease	-0.08	Decrease
15	rs1397705319	R144W	-1.273	Decrease	-1.44	Decrease
16	rs1401007590	C181W	-0.484	Decrease	0.45	**Increase**
17	rs1554626603	Y219S	-1.327	Decrease	-1.95	Decrease
18	rs1554626657	V151G	-1.814	Decrease	-2.61	Decrease
19	rs1554626663	P149L	-0.5223	Decrease	-0.01	Decrease
20	rs1554626665	Y147C	-1.567	Decrease	1.36	**Increase**
21	rs1554626675	N140I	-0.539	Decrease	1.76	**Increase**
22	rs1554626735	S68L	-0.142	Decrease	-0.36	Decrease
23	rs1554626749	A33T	-1.70	Decrease	-0.97	Decrease
24	rs1554626750	P27R	-0.465	Decrease	-1.65	Decrease
25	rs1554626752	P27S	-0.753	Decrease	-1.73	Decrease
26	rs1554626763	W17C	-0.926	Decrease	-1.44	Decrease
27	rs781905709	G177S	-0.788	Decrease	-0.96	Decrease
28	rs113016282	I21N	-1.28	Decrease	-1.24	Decrease
29	rs782101922	G100C	0.320	**Increase**	-1.29	Decrease

### Prediction of the molecular mechanism of pathogenicity

The MutPred2 server was used to evaluate the pathogenicity mechanisms of the core nsSNPs A78V, N140I, C181W, G100C, Y147C, and A128P. These nsSNPs demonstrated functional effects, such as loss/gain of regulatory binding interface, loss of relative solvent accessibility (RSA), altered transmembrane protein, altered metal binding, and loss of sulfation. A regulatory binding interface is a site on a protein where another molecule binds to control its activity by either activating or inhibiting it. The loss of the regulatory binding interface at position W137, caused by the mutation N140I, resulted in a significant alteration in the protein’s ability to regulate activation or inhibition. Whereas the gain of regulatory interface at F146 by the mutation Y147C will lead to unintended binding, resulting in a side effect on protein activity. The RSA of a protein residue measures its exposure to the solvent. A loss of RSA at a mutant residue can destabilize it by disrupting hydrogen bonds, salt bridges, electrostatic interactions, and other non-covalent interactions. Transmembrane proteins, which directly interact with the phospholipid bilayer, act as bridges for various molecules and ions and mediate activation responses to extracellular stimuli. The A128P and G100C nsSNPs show statistically significant potential to cause alterations in the transmembrane region. The C181W mutation may alter metal binding. These SNPs may disrupt the structure and function of metalloproteins, potentially leading to various diseases. A detailed description of nsSNPs with their mutant impact, including p-values and prediction scores, is provided in **Table 3**.

**Table 3:** Effect of CLDN-3 core SNPs on its function predicted by MutPred2.

S.No.	nsSNP ID	Amino acid mutation	MutaPred2 score	Effect	P-value
1.	rs1401007590	C181W	0.923	Altered metal binding	4.7E-03
				Gain of strand	0.05
2.	rs1199877723	A128P	0.858	Altered transmembrane protein	0.01
				Loss of helix	0.03
3.	rs1243569776	A78V	0.674	Loss of relative solvent accessibility	0.02
4.	rs1554626675	N140I	0.804	Loss of relative solvent accessibility	8.20E-04
				Loss of regulatory binding interface at W137	8.20E-04
5.	rs1554626665	Y147C	0.862	Gain of regulatory binding interface at F146	0.02
				Loss of sulfation at Y147	0.04
6.	rs782101922	G100C	0.941	Altered transmembrane protein	1.9E-04
				Gain of GPI-anchor amidation at N105	0.02

### Structural analysis

#### Homology modelling and model validation

**Figure 3:**
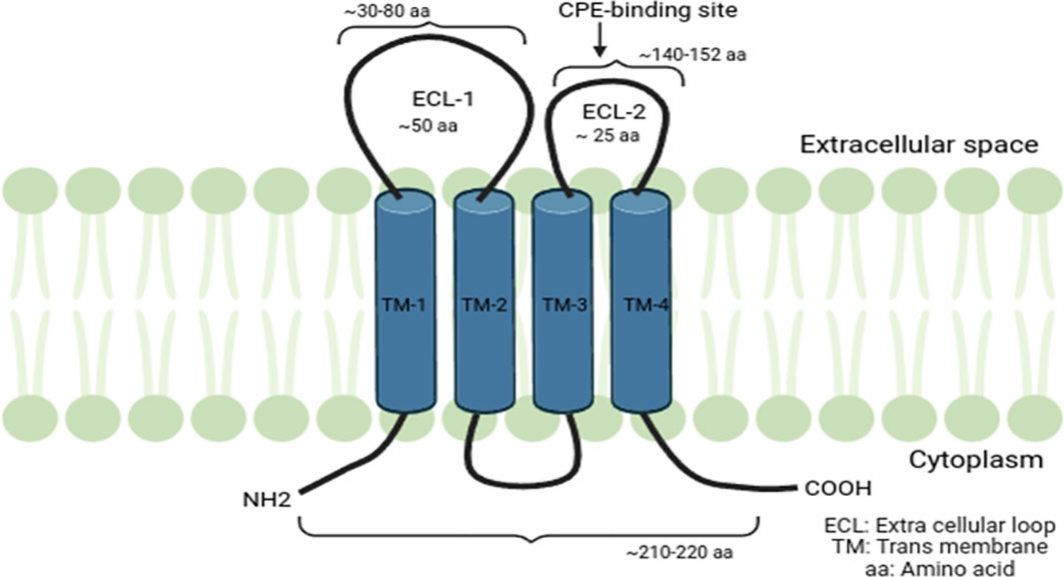
Structural models of CLDN-3 protein (adapted and modified from Yosuke et al., 2018) serve as a foundational model for understanding the structural impact of nsSNPs on these proteins.

The wild-type *CLDN-3*, along with its six core-nsSNPs (A78V, N140I, C181W, Y147C, and G100C), was structurally analysed using the SWISS-MODEL server. The template with ID 6AKE, exhibiting >90% sequence identity across all variants, was selected based on acceptable QMEAN and GMQE scores, as detailed in the **Supplementary Table 3**. Template 6AKE corresponds to the crystal structure of *CLDN-3* in mouse in complex with the C-terminal fragments of CPE, which has high structural and biological relevance to human *CLDN-3*. It provides a detailed resolution of CLDN-3-specific transmembrane and extracellular domains, which are directly involved in TJ morphology and CPE binding. A modelled structure of *CLDN-3* is shown in **Figure 3**, which serves as a foundational model for understanding the structural impact of nsSNPs on these TJ proteins. The homology-based modelled structures were validated for accuracy using PROCHECK, which ensures correct stereochemistry via Ramachandran plots. These plots show residues in the core and allowed regions, confirming the validity of the structures. For detailed visualization, please refer to **Supplementary Figure 2**.

#### RMSD value and TM align value

The RMSD of mutant residues for all the core-nsSNPs was calculated using PyMOL to assess structural similarity by superimposing them with the native protein. The TM-align value was also checked: a score of 1 indicates no dissimilarity, below 0.2 means unrelated structures, and above 0.5 indicates the same fold, as shown in **Table 4**.

**Table 4:** Structural analysis of CLDN-3 core-nsSNPs.

Amino acid mutation	TM align value	RMSD value	Residue in core region (Procheck)
C181W	0.947	0.769	90.1%
A128P	0.956	0.052	90.0%
A78V	0.995	0.052	90.7%
N140I	0.995	0.032	90.7%
Y147C	0.961	0.639	90.7%
G100C	0.952	0.701	90.7%

#### Chemical properties analysis

BIOVIA Discovery Studio Visualizer was employed to analyse the chemical properties for all core-nsSNPs. This analysis included assessing hydrophobicity, examining secondary structure, and comparing the number of hydrogen bonds formed by mutant residues with those in the native protein. On the hydrophobicity scale, higher positive values indicate greater hydrophobicity of an amino acid. The hydrophobic nature of the amino acid R groups causes them to cluster together in the interior region of a protein. This clustering is essential for stabilizing the protein structure and maintaining its overall shape. All mutants exhibit significant hydrophobicity, as detailed in **Table 5**.

**Table 5:** Chemical analysis of CLDN-3 core-nsSNPs by BIOVIA discovery studio visualizer.

S.N.	nsSNPs ID	Amino acid position		Residue	Hydrophobicity	Secondary structure	H- Bond
1	rs11549498	C181W	Native	Arginine	-4.5	Sheet	5
			Mutant	Cysteine	2.5	Sheet	2
2	rs781999702	A128P	Native	Leucine	3.8	Helix	3
			Mutant	Proline	-1.6	Helix	2
3	rs1243569776	A78V	Native	Alanine	1.8	Helix	2
			Mutant	Valine	4.2	Helix	3
4	rs1554626675	N140I	Native	Asparagine	-3.5	Coil	3
			Mutant	Isoleucine	4.5	Coil	3
5	rs1554626665	Y147C	Native	Tyrosine	2.5	Turn	3
			Mutant	Cysteine	-1.3	Turn	2
6	rs782101922	G100C	Native	Glycine	2.5	Helix	2
			Mutant	Cysteine	-1.3	Helix	5

#### Protein-protein docking analysis

Protein-protein docking is a computational approach used to predict the 3-D structure of protein complexes from the individual component structures, thereby facilitating the understanding of protein interactions and their functional implications. Instead of directly estimating binding affinity, docking platforms such as the HADDOCK server evaluate interactions using the HADDOCK score, a weighted scoring function that integrates various energetic terms, including van der Waals, electrostatic, and desolvation energies. In this context, more negative HADDOCK scores indicate more favourable and stable interactions.

In this study, to assess the interaction between ZO-1 (PDB ID: 2H3M) and *CLDN-3*, along with its six core-nsSNPs, protein-protein docking was performed using the HADDOCK server. The docking results (**Table 6**; **Supplementary Figure 3**) showed that the wild-type *CLDN-3* exhibits a relatively more favourable HADDOCK score with ZO-1 compared to most of its nsSNP variants, with the exception of the C181W mutant, which demands the need for further experimental validation. Mutations in *CLDN-3* that alter interaction strength, either weakening or excessively strengthening binding, may disrupt TJ integrity, potentially leading to aberrant activation of signalling pathways (e.g., Wnt, Akt), thereby promoting cell proliferation and EMT, which are critical processes in epithelial tumorigenesis (**Supplementary Figure 4**).

**Table 6:** Docking analysis of CLDN-3 with ZO-1 along with its core-nsSNPs.

CLDN-3 wild-type with ZO-1 PDZ1	
HADDOCK score	-80.6 +/- 6.9
Cluster size	11
RMSD from the overall lowest-energy structure	2.6 +/- 2.1
Van der Waals energy	-30.7 +/- 3.6
Electrostatic energy	-259.2 +/- 40.0
Desolvation energy	0.9 +/- 1.0
Restraints violation energy	10.8 +/- 12.5
Buried Surface Area	1322.8 +/- 102.4
Z-Score	-2
A78V mutant with ZO-1 PDZ1	
HADDOCK score	-78.1 +/- 2.6
Cluster size	11
RMSD from the overall lowest-energy structure	10.9 +/- 0.1
Van der Waals energy	-30.7 +/- 2.4
Electrostatic energy	-269.4 +/- 51.8
Desolvation energy	4.5 +/- 6.5
Restraints violation energy	19.0 +/- 11.9
Buried Surface Area	1259.4 +/- 69.0
Z-Score	-1.9
N140I mutant with ZO-1 PDZ1	
HADDOCK score	-80 +/- 4.9
Cluster size	15
RMSD from the overall lowest-energy structure	1.1 +/- 0.7
Van der Waals energy	-42.5 +/- 2.8
Electrostatic energy	-214.9 +/- 25.7
Desolvation energy	3.2 +/- 1.2
Restraints violation energy	16.9 +/- 14.4
Buried Surface Area	1287.1 +/- 64.3
Z-Score	-2.2
A128P mutant with ZO-1 PDZ1	
HADDOCK score	-67.1 +/- 4.2
Cluster size	76
RMSD from the overall lowest-energy structure	10.0 +/- 0.2
Van der Waals energy	-47.8 +/- 4.4
Electrostatic energy	-88.2 +/- 13.2
Desolvation energy	-2.0 +/- 2.3
Restraints violation energy	3.3 +/- 1.0
Buried Surface Area	1125.0 +/- 73.2
Z-Score	-1.2
G100C mutant with ZO-1 PDZ1	
HADDOCK score	-58.3 +/- 0.6
Cluster size	46
RMSD from the overall lowest-energy structure	3.3 +/- 0.1
Van der Waals energy	-38.2 +/- 2.3
Electrostatic energy	-114.0 +/- 14.6
Desolvation energy	2.4 +/- 3.9
Restraints violation energy	3.3 +/- 1.9
Buried Surface Area	956.0 +/- 39.4
Z-Score	-1.4
C181W mutant with ZO-1 PDZ1	
HADDOCK score	-85.2 +/- 8.7
Cluster size	19
RMSD from the overall lowest-energy structure	0.7 +/- 0.4
Van der Waals energy	-60.4 +/- 2.5
Electrostatic energy	-150.9 +/- 27.3
Desolvation energy	1.8 +/- 1.4
Restraints violation energy	34.8 +/- 18.8
Buried Surface Area	1376.2 +/- 75.9
Z-Score	-2.5
Y147C mutant with ZO-1 PDZ1	
HADDOCK score	-62.5 +/- 1.4
Cluster size	115
RMSD from the overall lowest-energy structure	0.7 +/- 0.5
Van der Waals energy	-44.4 +/- 2.8
Electrostatic energy	-98.4 +/- 33.9
Desolvation energy	1.2 +/- 3.5
Restraints violation energy	3.5 +/- 0.8
Buried Surface Area	1020.6 +/- 48.2

#### MD simulation analysis

To identify the structural consequences of mutations at the atomic level and in a dynamic context, MD simulations were performed for wild-type CLDN-3 and its respective nsSNPs. Seven systems were analysed for *CLDN-3* (wild-type, A78V, C181W, N140I, A128P, Y147C, and G100C); each system was simulated for 100 ns using GROMACS, and all 10,000 frames from the 100 ns of simulation were recorded for analysis.

#### RMSD analysis

**Figure 4:**
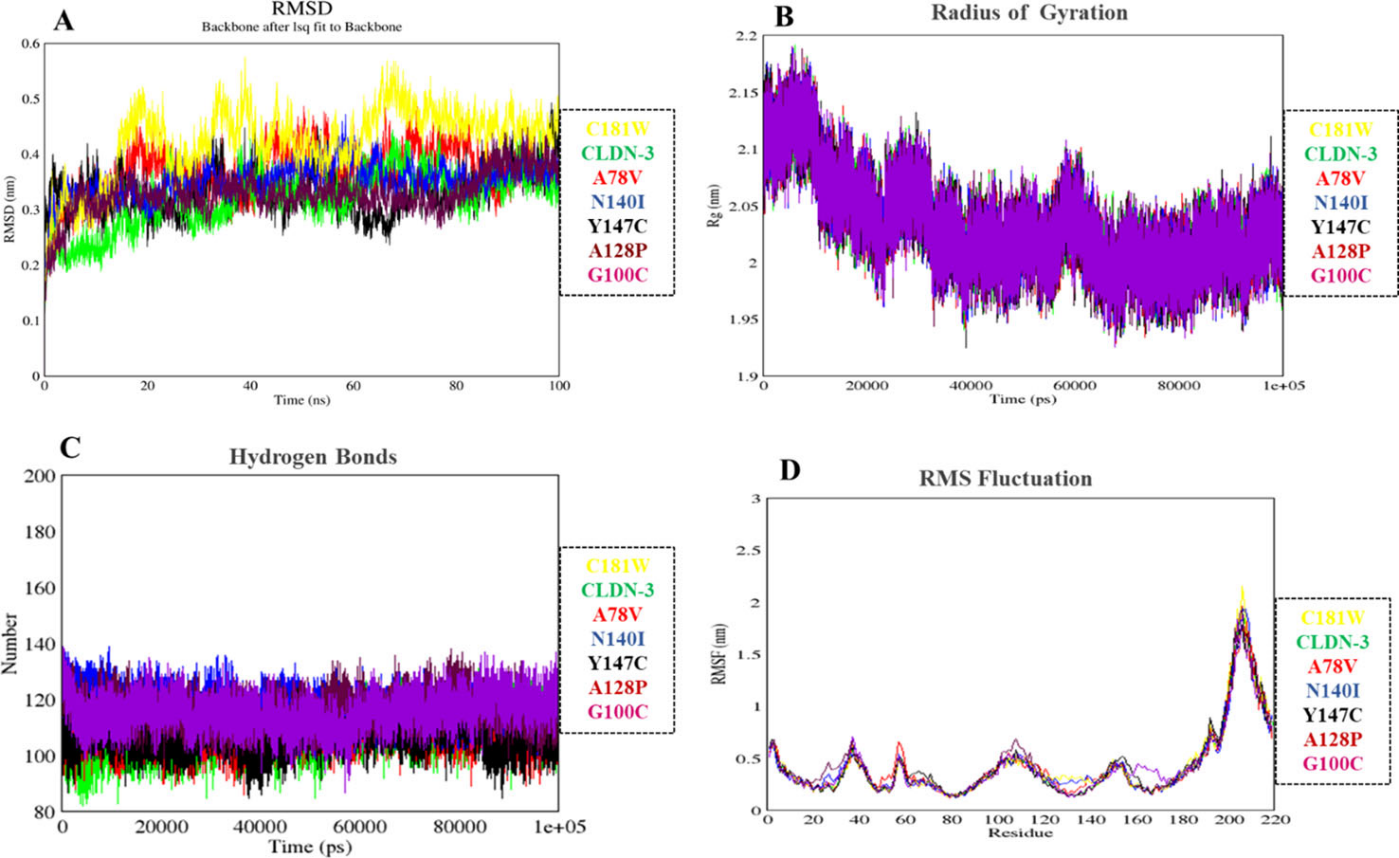
Molecular dynamic simulation analysis for CLDN-3 along with its core-nsSNP A78V, N140I, C181W, A128P, Y147C, and G100C. **(A)** RMSD analysis, which is represented as time-dependent fluctuation during the simulation, **(B)** Rg analysis, which is represented as time-dependent flexibility during the analysis, **(C)** H-bond analysis, which shows time-dependent H-bond formation during the simulation, and **(D)** RMSF analysis, which is represented as residue-dependent fluctuation during the simulation.

RMSD was calculated for all seven protein systems to evaluate their structural stability. This parameter is widely used to assess global deviations in the protein backbone throughout the simulation. Notably, higher RMSD deviations in mutant structures imply that the corresponding amino acid substitutions may weaken intramolecular interactions essential for maintaining *CLDN-3* structural integrity. Such destabilization could adversely affect the protein’s role within TJ complexes, where conformational stability is crucial for proper junction assembly and barrier function. Although some mutant systems displayed RMSD profiles comparable to the wild type, their distinct fluctuation patterns indicate subtle but persistent conformational rearrangements that may still perturb native *CLDN-3* activity. The comparative RMSD versus time plots in **Figure 4A** highlight stability differences between wild-type and mutant CLDN-3 systems over the simulation timeframe.

#### Rg analysis

The Rg analysis of a system reflects the root-mean-square distance of the protein’s atoms from the axis of rotation and is a useful measure of protein structure compactness. The plots in **Figure 4B** showing the variation in Rg over time for CLDN-3 demonstrate that all systems exhibit high compactness. A lower Rg value in the mutant structure suggests a more compact, potentially more stable conformation, which might result in reduced flexibility and enhanced structural rigidity; it could also hinder conformational transitions necessary for *CLDN-3*’s dynamic interactions in TJ assembly.

#### H-bond analysis

Hydrogen bonds are crucial for maintaining protein structural stability. A higher average number of hydrogen bonds indicates a more stable protein structure, while a lower number suggests reduced stability. The data, presented in **Figure 4C** as well as in **Supplementary Table 4** shows the number of intramolecular hydrogen bonds over time for CLDN-3, including the mutations, highlighting their high stability. So, excessive rigidity due to increased H-bonds may reduce CLDN-3’s adaptability during TJ formation, potentially impairing its biological function in TJ dynamics.

#### RMSF analysis

RMSF analysis highlighted notable differences in residue-level flexibility between the wild-type and mutant *CLDN-3* structures. Consistent with protein architecture, loop, turn, and coil regions exhibited higher fluctuations than α-helices and β-sheets; however, several mutants showed increased RMSF peaks relative to the wild type, indicating mutation-induced enhancement of local flexibility. Importantly, these highly dynamic regions overlap with functionally relevant domains, including extracellular loops and sites involved in partner-protein interactions. Elevated flexibility in these regions may weaken *CLDN-3* interactions within TJ complexes or alter the CPE binding interface, thereby affecting junction assembly and permeability. The residue-wise RMSF profiles shown in **Figure 4D** demonstrate that these effects are localized rather than global, suggesting targeted functional disruption rather than complete structural destabilization.

#### RegulomeDB annotation analysis of non-coding SNPs

**Figure 5:**
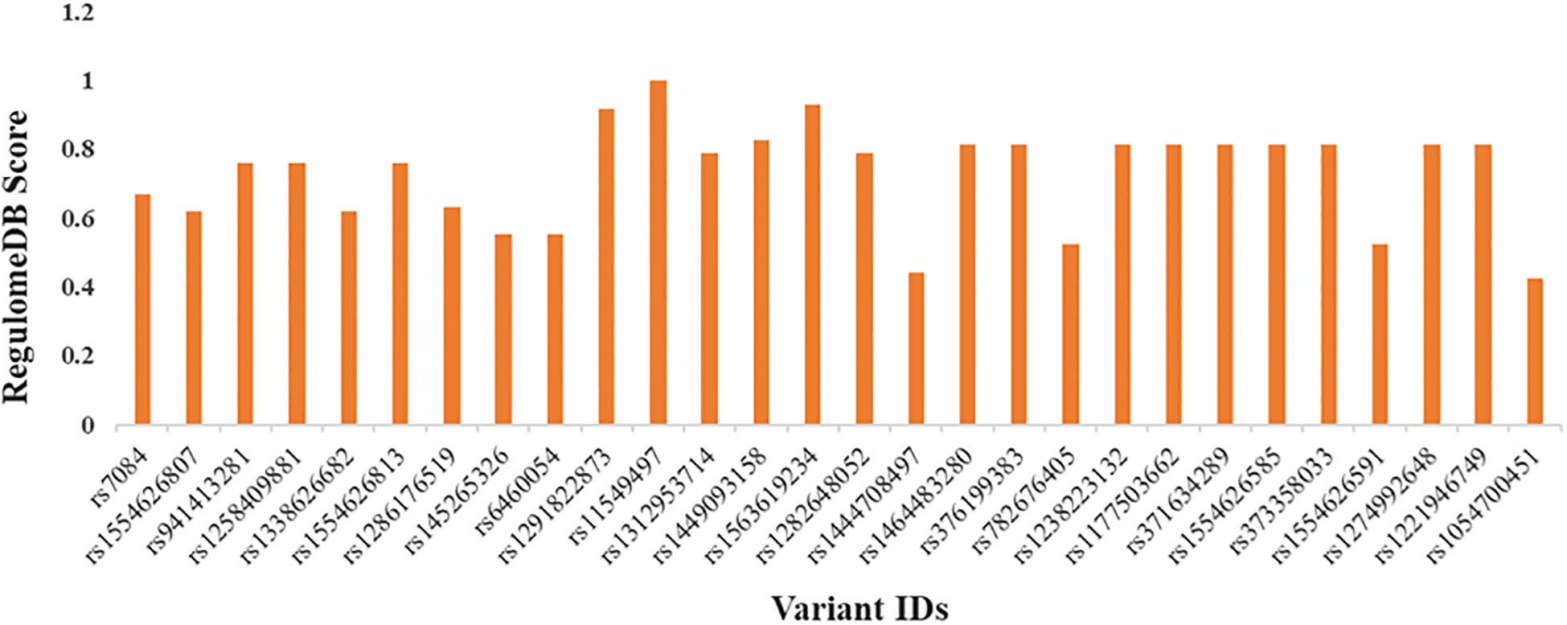
Annotation and scoring of 28 UTR SNPs obtained through the RegulomeDB database.

From the Ensembl database, we retrieved a total of 150 3’UTR SNPs and 106 5’UTR SNPs (**Supplementary Table 2** and **Supplementary Figure 1**). To identify impactful non-coding SNPs among the retrieved UTR SNPs, annotation was performed using the RegulomeDB database. Based on the scoring, we found 13 5’UTR SNPs out of 106 and 15 3’ UTR SNPs out of 150 found to be significantly scored<= 2b. Significantly annotated 28 UTR SNPs with their score in detail are shown in **Table 7**, **Figure 5**.

**Table 7:** List of regulome DB annotated SNPs.

S. N.	SNPs ID	Allele	Probability	Ranking	Types
1.	rs7084	G/A/C/T	0.66703	1f	5’ UTR
2.	rs1554626807	G/C	0.61749	2b	5’ UTR
3.	rs941413281	C/A	0.76166	2b	5’ UTR
4.	rs1258409881	G/A/T	0.76166	2b	5’ UTR
5.	rs1338626682	A/G	0.61749	2b	5’ UTR
6.	rs1554626813	G/T	0.76166	2b	5’ UTR
7.	rs1286176519	G/A/C	0.63284	2b	5’ UTR
8.	rs145265326	G/A	0.55436	1f	5’ UTR
9.	rs6460054	T/C	0.55436	1f	5’ UTR
10.	rs1291822873	C/T	0.91667	2a	5’ UTR
11.	rs11549497	G/A/C	1	2a	5’ UTR
12.	rs1312953714	C/A	0.78848	2b	5’ UTR
13.	rs1449093158	T/C	0.82541	2b	5’ UTR
14.	rs1563619234	G/A/T	0.93104	2b	3’UTR
15.	rs1282648052	T/C	0.7889	2b	3’UTR
16.	rs1444708497	G/A	0.44059	2b	3’UTR
17.	rs1464483280	G/A/C	0.81166	2b	3’UTR
18.	rs376199383	T/A/G	0.81166	2b	3’UTR
19.	rs782676405	G/T	0.52283	2b	3’UTR
20.	rs1238223132	G/A	0.81166	2b	3’UTR
21.	rs1177503662	G/T	0.81166	2b	3’UTR
22.	rs371634289	G/A	0.81166	2b	3’UTR
23.	rs1554626585	G/T	0.81166	2b	3’UTR
24.	rs373358033	G/A	0.81166	2b	3’UTR
25.	rs1554626591	G/A	0.52283	2b	3’UTR
26.	rs1274992648	T/A/G	0.81166	2b	3’UTR
27.	rs1221946749	G/T	0.81166	2b	3’UTR
28.	rs1054700451	G/T	0.42482	2b	3’UTR

#### miRNA-related SNP analysis

Analysis of miRNA-associated SNPs within the *CLDN-3* 3′ UTR revealed that several variants have the potential to alter miRNA-mRNA interactions, thereby influencing post-transcriptional regulation. Among the 15 analysed 3′UTR SNPs, four variants (rs1282648052, rs1444708497, rs1464483280, and rs1054700451) were predicted to induce both miRNA target gain and loss, suggesting substantial remodelling of regulatory binding landscapes. In addition, four SNPs (rs1238223132, rs371634289, rs373358033, and rs1554626591) were associated exclusively with target gain, indicating the creation of novel miRNA binding sites that may suppress gene expression. These findings, summarized in **Table 8**, highlight specific 3′UTR variants that may modulate *CLDN-3* expression through altered miRNA regulation and warrant further functional validation.

**Table 8:** miRNAs-SNPs association analysis of 3’ UTR SNPs.

SNPs ID	Position	Allele	Target gain with SNP in 3’UTR	Target loss with SNP in 5’UTR
rs1282648052	chr7:73769331	T/C	2 (hsa-miR-8072, hsa-miR-3960)	1 (hsa-miR-579-5p)
rs1444708497	chr7:73769333	G/A	3 (hsa-miR-10395-5p, hsa-miR-8069, hsa-miR-598-5p)	1 (hsa-miR-6786-5p)
rs1464483280	chr7:73769341	G/A	4 (hsa-miR-142-3p, hsa-miR-4255, hsa-miR-4536-5p, hsa-miR-6073)	1 (hsa-miR-4694-5p)
rs1054700451	chr7:73769147	G/T	4 (hsa-miR-493-3p, hsa-miR-380-5p, hsa-miR-1304-5p, hsa-miR-563)	6 (hsa-miR-7515, hsa-miR-9986, miR-3179, miR-1294, miR-4316, miR-4710)

#### TFBS analysis of 5’ UTR SNPs

We observed no TFBS hits in our analysis using the TRANSFAC database and the SNP2TFBS web server. This absence could arise from multiple factors. Firstly, it may reflect a genuine biological absence of known TFBSs within the specific upstream regulatory regions we analysed for *CLDN-3*. Alternatively, the absence could result from tool-specific limitations, such as reliance on a curated database with incomplete entries.

#### eQTL analysis

**Figure 6:**
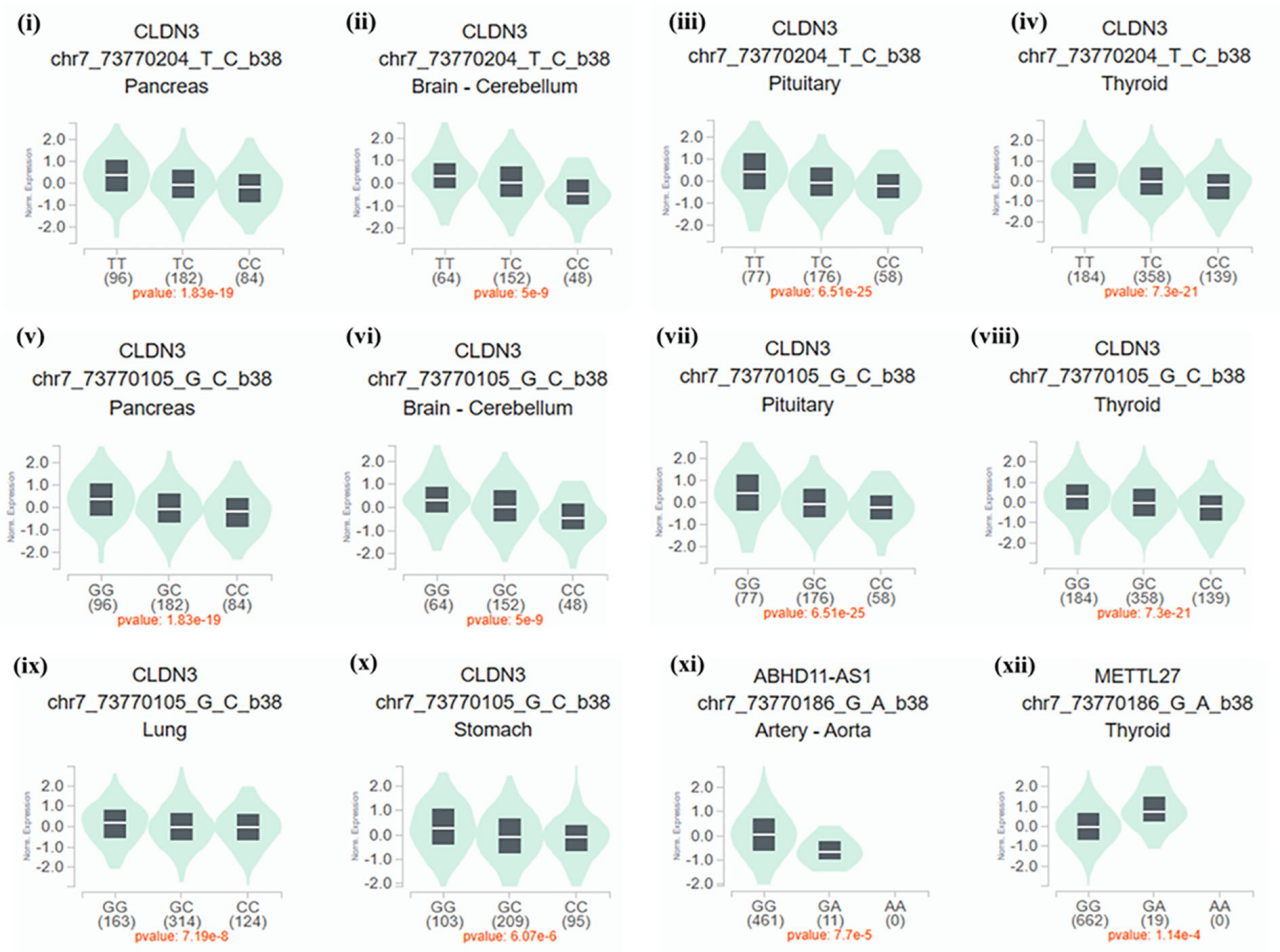
Single-Tissue eQTL Analysis of UTR using GTEx portal. Violin plots show the impact of SNP rs6460054 (i-iv), rs7084 (v-x), and rs145265326 (xi-xii) on CLDN-3 gene expression.

Through the analysis, we found that of 28 SNPs, only three were annotated in the GTEx portal for single-tissue eQTL analysis associated with *CLDN-3* in independent disease cases. As we can see, the SNP rs6460054 allele ‘C’ significantly affects the gene expression in pancreas, brain-cerebellum, pituitary, and thyroid disease cases. Similarly, the SNP rs7084 allele ‘C’ significantly regulates gene expression in the above-mentioned disease, including lung and stomach cases. Whereas SNP rs145265326 allele ‘A’ association with *CLDN-3* was not found, but its significantly strong association with *AS1* and *METTL27* genes has been found, as demonstrated in **Figure 6**.

#### RNA fold analysis

**Figure 7:**
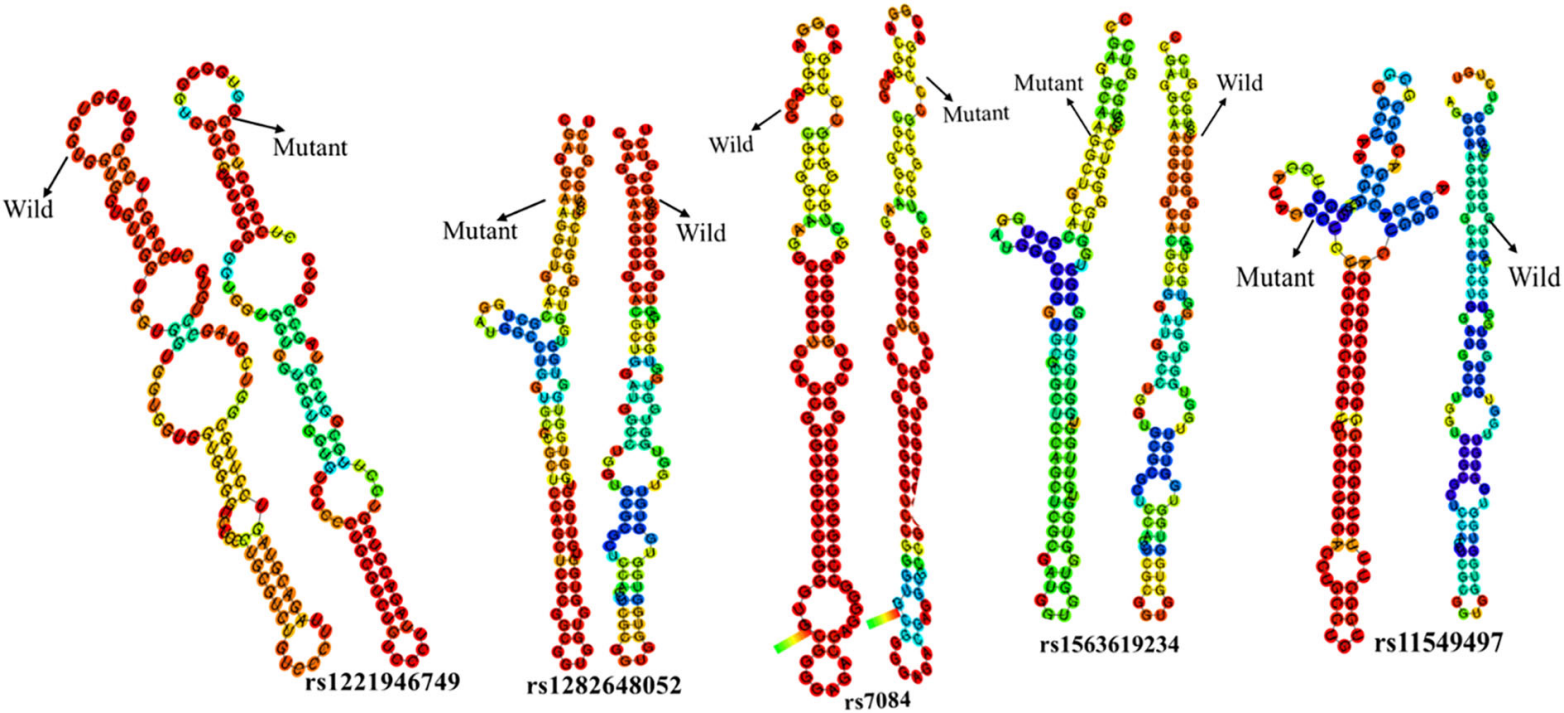
mRNA secondary structure comparison of UTR SNP variants and wild-type CLDN-3.Predicted secondary structures generated using RNAfold showing conformational differences between wild-type and selected stabilized UTR SNP variants, indicating the impact of SNPs on mRNA folding and structural stability.

To evaluate the functional impact of UTR variants on *CLDN-3* mRNA stability, MFE changes were analysed for SNPs located in untranslated regions. Among the 28 UTR SNPs examined, five variants (rs1282648052, rs1221946749, rs7084, rs11549497, rs1563619234) exhibited lower MFE values following mutation, indicating enhanced mRNA structural stability. Increased stability of UTR regions may influence post-transcriptional regulation by altering mRNA turnover, translational efficiency, or the binding of regulatory factors. The comparative MFE changes summarized in **Figure 7** and **Table 9** support the potential regulatory relevance of these variants. Given their stabilizing effect on mRNA structure, these SNPs may be promising candidates for further experimental validation as potential biomarkers in gynaecological and other epithelial cancers.

**Table 9:** Minimum free energy (MFE) analysis of wild and mutant structures of mRNAs caused by 3’, 5’ UTR SNPs.

S. N.	SNPs ID	Allele	Location (Chr 7)	Start region	End region	MFE wild (kcal/mol)	MFE mutated (kcal/mol)
1	rs1054700451	G/T	73769147	73769097	73769199	-32.3	-30.8
2	**rs1563619234**	**G/A/T**	**73769330**	**73769280**	**73769380**	**-33.3**	**-35.00**
3	**rs1282648052**	**T/C**	**73769331**	**73769281**	**73769381**	**-34.4**	**-38.4**
4	rs1444708497	G/A	73769333	73769283	73769383	-30.8	-30.4
5	rs1464483280	G/A/C	73769341	73769291	73769391	-29.7	-29.9
6	rs376199383	T/A/G	73769345	73769295	73769395	-32.1	-30.60
7	rs782676405	G/T	73769348	73769298	73769398	-34.7	-34.2
8	rs1238223132	G/A	73769350	73769300	73769400	-34.7	-35.6
9	rs1177503662	G/T	73769351	73769301	73769401	-33.2	-32.1
10	rs371634289	G/A	73769356	73769306	73769406	-33.3	-34.6
11	rs1554626585	G/T	73769357	73769307	73769407	-32.9	-31.4
12	rs373358033	G/A	73769359	73769309	73769409	-31.9	-32.9
13	rs1554626591	G/A	73769363	73769313	73769413	-32.7	-31.3
14	rs1274992648	T/A/G	73769364	73769314	73769414	-33.7	-33.3
15	**rs1221946749**	**G/T**	**73769367**	**73769317**	**73769417**	**-22.7**	**-27.2**
16	**rs7084**	**G/A/C/T**	**73770105**	**73770055**	**73770155**	**-53.4**	**-55.2**
17	rs1554626807	G/C	73770148	73770098	73770198	-42.6	-41.1
18	rs941413281	C/A	73770150	73770100	73770200	-42.5	-43.3
19	rs1258409881	G/A/T	73770151	73770101	73770201	-42.1	-43.1
20	rs1338626682	A/G	73770153	73770103	73770203	-42.5	-47.2
21	rs1554626813	G/T	73770155	73770105	73770205	-44.8	-44.2
22	rs1286176519	G/A/C	73770163	73770113	73770213	-37.6	-35.9
23	rs145265326	G/A	73770186	73770136	73770236	-32.5	-32.2
24	rs6460054	T/C	73770204	73770154	73770254	-35.4	-35.4
25	rs1291822873	C/T	73770217	73770167	73770267	-43	-42.1
26	**rs11549497**	**G/A/C**	**73770218**	**73770168**	**73770268**	**-43.7**	**-48**
27	rs1312953714	C/A	73770236	73770186	73770286	-55.7	-53.5
28	rs1449093158	T/C	73770244	73770194	73770294	-57	-57

## Discussion

Over the years, advancements in sequencing technologies have generated vast amounts of multi-omics data, including genomics, transcriptomics, and proteomics, which have been deposited in public repositories worldwide (47). This abundance of publicly available raw data has enabled researchers to explore multiple research gaps from diverse perspectives, facilitating extensive secondary analyses using a wide range of computational approaches. This study focuses on an integrative *in silico analysis of coding and non-coding SNPs in the human CLDN-3 gene, aiming to identify high-priority variants that may serve as biomarkers* for epithelial cancers. *CLDN-3* is a tight junction protein essential for epithelial barrier integrity and cell polarity. Dysregulation of *CLDN-3* has been widely reported in gynaecological and other epithelial cancers, making it an attractive biomarker and therapeutic target, particularly in the context of CPE-based therapies (7, 8, 10, 11). For the current study, methodology was divided into two major analytical arms: (i) coding SNP analysis to assess structural and functional protein alterations, and (ii) non-coding SNP analysis to evaluate regulatory effects on gene expression and mRNA stability (**Figure 1**). A total of 1,578 SNPs associated with *CLDN-3* were retrieved from the dbSNP and Ensembl. These included 140 nsSNPs, 150 SNPs in the 3′UTR, and 106 SNPs in the 5′UTR (**Supplementary Table 1**, **2** & **Supplementary Figure 1**), necessitating stringent prioritization strategies. Using SIFT, PolyPhen-2, and SNPs&GO, 29 nsSNPs (**Table 1**) were consistently predicted as deleterious out of 140 nsSNPs. This consensus-based filtering increases confidence in the biological relevance of the shortlisted variants. Since proteins don’t function in isolation within the cell, they work in a network (47). The PPI analysis revealed that *CLDN-3* interacts strongly with TJ components, including ZO-1, OCLN, and other claudins (**Figure 2**). This highlights that mutations in *CLDN-3* could propagate instability across the TJ complex. Further to assess the impact of mutation on consensus nsSNPs, I-Mutant 2.0 and MUpro web server were used. Stability predictions identified six core nsSNPs (A78V, G100C, A128P, N140I, Y147C, C181W) that showed increased stability (**Table 2**). While 23 nsSNPs (R30C, P134Q, I143N, D75H, L15P, C24G, L129R, Q62R, V107E, V119E, G93R, K64E, R144W, Y219S, V151G, P149L, S68L, A33T, P27R, P27S, W17C, G177S, I21N) showed decreased stability (**Table 2**), and any alteration in protein stability may cause misfolding, degradation or aberrant oligomerisation of proteins (1, 48). Therefore, for further functional analyses, we considered nsSNPs associated with increased protein stability. Despite enhanced stability, such variants may remain pathogenic by inducing excessive structural rigidity or by altering membrane dynamics. The MutPred2 tool was used to classify missense mutations as pathogenic or benign. The predicted pathogenic properties include changes in protein stability, disruption of binding sites, and alterations in post-translational modification sites, and it provides pathogenicity probability along with a statistically significant p-value. MutPred2 analysis suggested that these variants may perturb regulatory interfaces, transmembrane behaviour, binding properties, and several other functional features, as they are showing significant statistical association with predicted properties (**Table 3**). Notably, N140I and Y147C were predicted to affect regulatory binding sites near the CPE interaction domain. Moreover, for structural analysis, homology models were generated using SWISS-MODEL and validated through Ramachandran plot analysis, confirming the stereochemical reliability of both wild-type and mutant structures (**Supplementary Figure 2**). RMSD and TM-align analyses showed conserved global folds across mutants (**Table 4**), but local structural perturbations were evident, particularly near functionally important regions. Mutations altered hydrophobicity and hydrogen bonding patterns, which are critical for transmembrane proteins like *CLDN-3* and may influence membrane embedding and extracellular interactions (**Table 5**). Protein-protein docking revealed altered binding affinities between mutant *CLDN-3* proteins and ZO-1 (**Table 6**; **Supplementary Figure 3**). The C181W mutation showed enhanced binding, suggesting potential abnormal complex formation. MD simulations over 100 ns demonstrated mutation-induced changes in stability, compactness, flexibility, and hydrogen bonding, supporting the functional impact of the identified nsSNPs (**Figures 4A–D**). RegulomeDB analysis prioritized 28 regulatory SNPs (13 in the 5′ UTR and 15 in the 3′ UTR) with high regulatory potential (**Table 7** & **Figure 5**). Four 3′UTR SNPs exhibited both miRNA target gain and loss, indicating significant post-transcriptional regulatory effects (**Table 8**). No TFBS disruptions were detected. This may reflect true biological absence or limitations in available TF motif databases. The eQTL analysis in this study was performed using the GTEx web portal, which provides comprehensive gene expression data across multiple human tissues. GTEx analysis identified SNPs influencing *CLDN-3* expression across multiple tissues (**Figure 6**). However, these findings are tissue-based rather than cancer-specific, and sample sizes vary substantially across tissues. Notably, gynaecological tissues such as the ovary, cervix, and uterus are relatively underrepresented compared with tissues like the brain, blood vessels, and adipose tissue. Consequently, the non-coding SNPs of *CLDN-3* did not yield any tissue-specific regulatory hits relevant to gynaecological cancers. RNA fold analysis showed that several SNPs altered minimum free energy, suggesting changes in mRNA stability and regulatory accessibility (**Figure 7**, **Table 9**). Any mutation in *CLDN-3* that results in either weakened or excessively strengthened binding affinity may disrupt the formation of homogeneous tight junctions. Such perturbations can subsequently activate oncogenic signalling pathways, including Wnt/β-catenin, PI3K/Akt, and others (6, 16). Thereby promoting cell proliferation and EMT, which are key processes in the initiation and progression of epithelial neoplasms (**Supplementary Figure 4**). All these prioritized coding and non-coding *CLDN-3* variants identified through regulatory and structural analyses were subsequently queried against the ClinVar (49) database to assess their reported associations with cancer susceptibility or drug response. Notably, none of these variants showed documented clinical associations, indicating that they are currently unreported or underexplored in clinical datasets. This absence of annotation highlights the novelty of the identified variant set and supports their classification as high-priority coding and non-coding *CLDN-3* variants with potential to influence protein function, gene regulation, and therapeutic response. These findings underscore the importance of further experimental and clinical investigations to establish their relevance in cancer biology. These findings are computational predictions and require experimental validation, including mutagenesis, binding assays, functional tight junction studies, and cancer-specific expression analyses.

## Conclusion

Abnormal expression of the *CLDN-3* gene across various gynaecological cancers and other epithelial neoplasms makes the analysis of their coding and non-coding SNPs a promising approach for identifying high-priority variants. In this current *in-silico* SNPs analysis study, we found six core-nsSNPs, A78V, C181W, N140I, A128P, Y147C, and G100C that were significantly associated with *CLDN-3*, exhibiting a substantial functional impact. Notably, the mutations N140I and Y147C in CLDN-3 are located near the CPE-binding region, which is critical for the efficacy of CPE-based therapies targeting cancer cells overexpressing *CLDN-3*. Structural alterations caused by these mutations could impair or modify CPE binding affinity, potentially affecting the therapeutic response. This insight highlights the potential utility of N140I and Y147C as high-priority variants for patient stratification and drug-sensitivity profiling in clinical settings. Four 3’ UTR SNPs, rs1282648052, rs1444708497, rs1464483280, and rs1054700451, were found to be associated with miRNA, showing both target gain and loss properties. Three 5’ UTR SNPs, rs6460054, rs7084, and rs145265326, represent impactful distribution across different genotypes associated with *CLDN-3*. Whereas five SNPs, rs1282648052, rs1221946749, rs7084, rs11549497, and rs1563619234, have a significant effect on its secondary mRNA structure. Unlike earlier studies focusing solely on *CLDN-3* expression in cancer, our work provides the first comprehensive analysis of nsSNPs and regulatory SNPs, linking them to structural and functional disruptions. Although our *in-silico* analysis provides valuable predictions of SNP functional impact, such approaches are inherently limited by computational assumptions and database coverage. So, these *in silico-*identified SNPs might serve as high-priority variants requiring further experimental validation for future clinical applications. Future validation should include site-directed mutagenesis, binding assays to assess interaction with CPE, and *in vitro* assays to evaluate the biological impact of key mutations on TJ formation and drug response.

## Data Availability

The original contributions presented in the study are included in the article/**Supplementary Material**. Further inquiries can be directed to the corresponding author.
