# Supplementary figures and images for: An integrative coding and non-coding SNPs analysis of the CLDN-3 gene in humans to identify high-priority variants using in-silico analysis
Source: Front Oncol. 2026 Jun 29;16:1816524. doi: 10.3389/fonc.2026.1816524 (PMC13357936; doi:10.3389/fonc.2026.1816524)

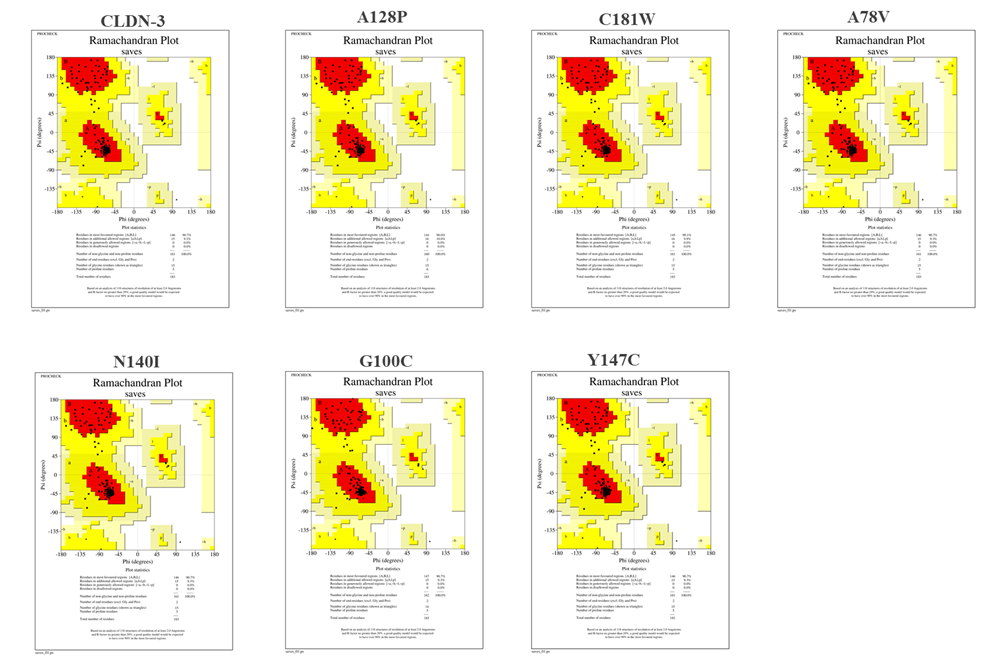

Supplement: Supplementary file 2 [file Image1.tif]

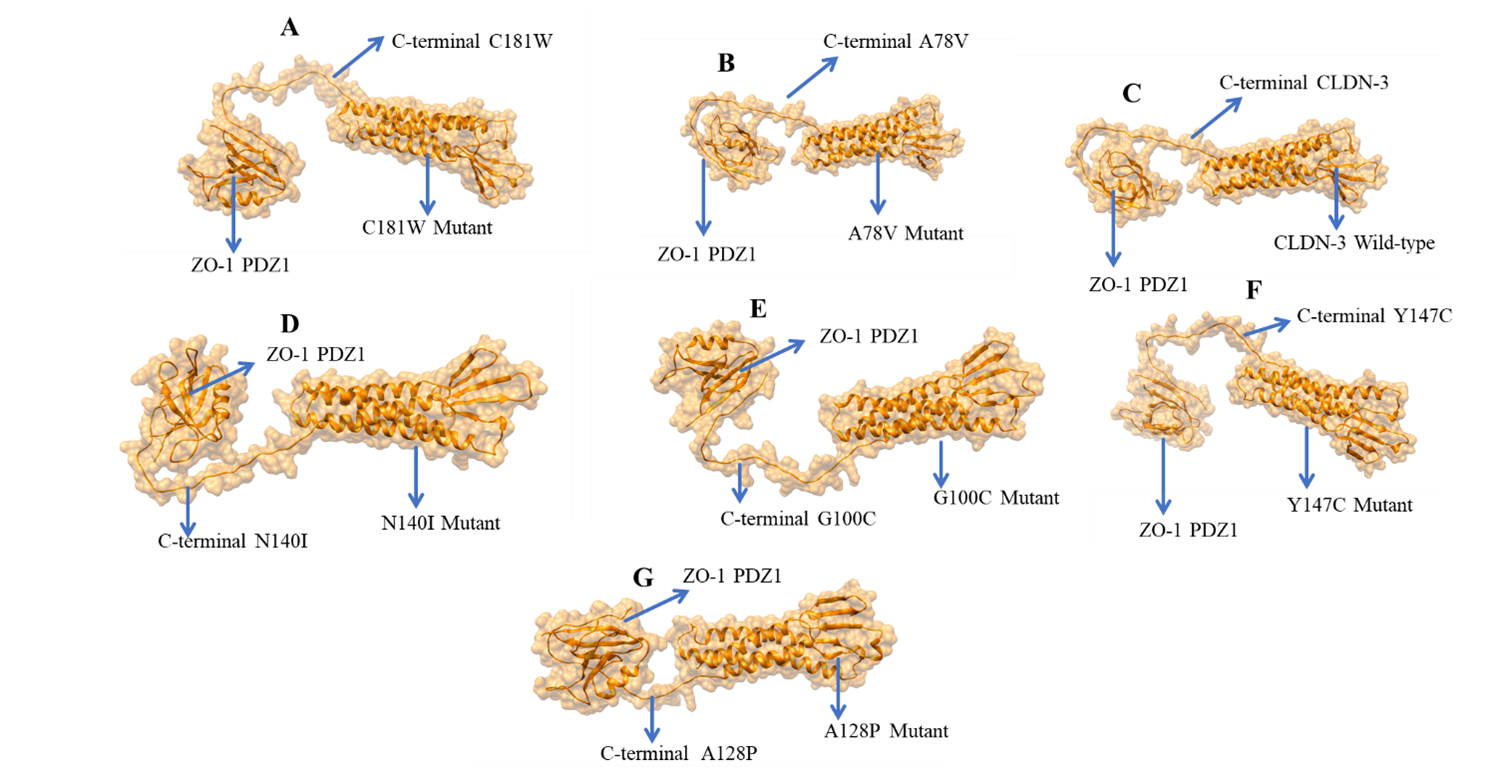

Supplement: Supplementary file 3 [file Image2.tif]

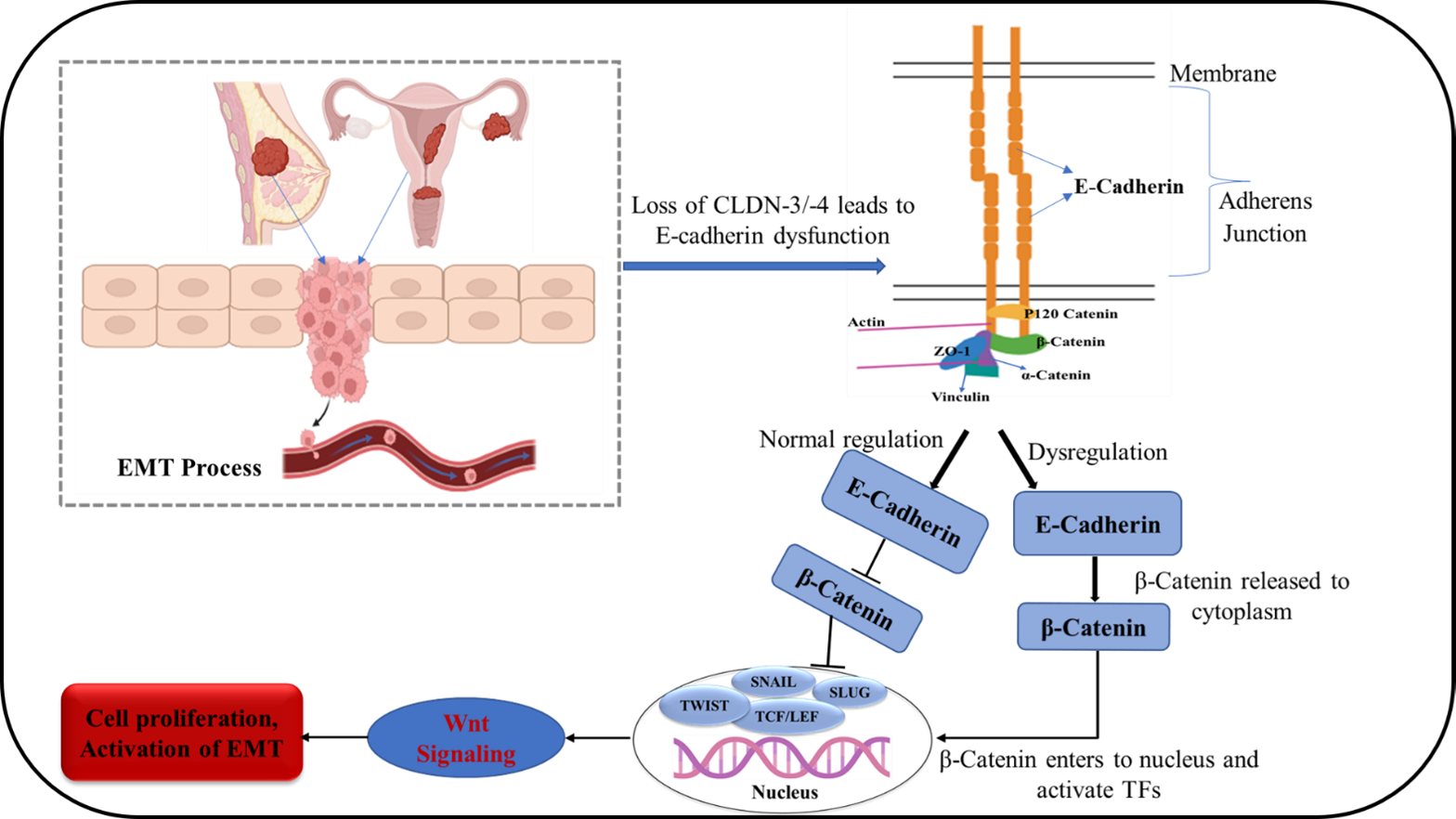

Supplement: Supplementary file 4 [file Image3.tif]

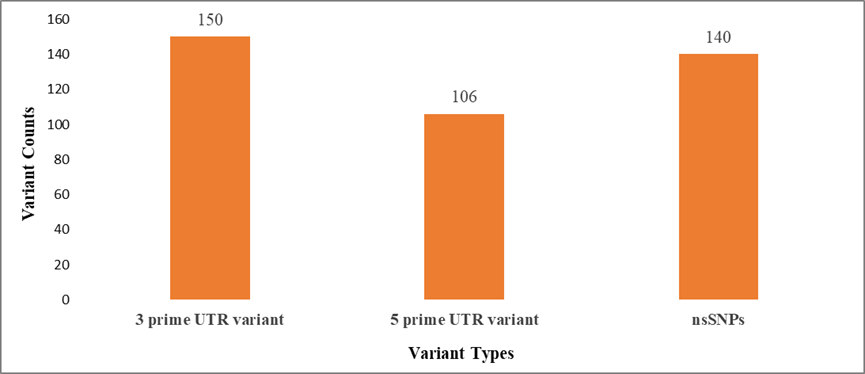

Supplement: Supplementary file 5 [file Image4.tif]
