# Supplementary material for: An integrative coding and non-coding SNPs analysis of the CLDN-3 gene in humans to identify high-priority variants using in-silico analysis
Source: Front Oncol. 2026 Jun 29;16:1816524. doi: 10.3389/fonc.2026.1816524 (PMC13357936; doi:10.3389/fonc.2026.1816524)
Supplement: Supplementary file 6 [file Table1.docx]

Suppl. Table 2: List of 140 coding SNPs predicted by different prediction tools for CLDN-3

| S. N0. | nsSNP ID | Amino acid change | SIFT-Score | SIFT- Prediction | PolyPhen2 score | PolyPhen2  prediction | SNP  &GO Index | SNP&  GO |
| --- | --- | --- | --- | --- | --- | --- | --- | --- |
| 1 | rs9133 | A112S | 0.32 | Tolerated | 0 | Benign | 6 | Neutral |
| 2 | rs11549498 | R30C | 0 | Deleterious | 1 | Damaging | **4** | Disease |
| 3 | rs80267179 | I21V | 0.46 | Tolerated | 0.58 | Damaging | 9 | Neutral |
| 4 | rs113016282 | I21T | 0.01 | Deleterious | 1 | Damaging | 4 | Neutral |
| 5 | rs113016282 | I21N | 0 | Deleterious | 1 | Damaging | **1** | Disease |
| 6 | rs139191328 | P134Q | 0 | Deleterious | 1 | Damaging | **2** | Disease |
| 7 | rs141257286 | I143N | 0 | Deleterious | 0.988 | Damaging | **5** | Disease |
| 8 | rs149948419 | C24G | 0.03 | Deleterious | 1 | Damaging | 5 | Neutral |
| 9 | rs200567790 | M51I | 0 | Deleterious | 0.298 | Benign | 4 | Disease |
| 10 | rs200825628 | A97S | 0.57 | Tolerated | 0.001 | Benign | 8 | Neutral |
| 11 | rs201650771 | D75H | 0.04 | Deleterious | 1.000 | Damaging | **0** | Disease |
| 12 | rs370107198 | T192M | 0.22 | Tolerated | 0.005 | Benign | 10 | Neutral |
| 13 | rs371936142 | A114T | 0.01 | Deleterious | 0.095 | Benign | 4 | Neutral |
| 14 | rs372826102 | G4S | 0.04 | Deleterious | 0.907 | Damaging | 7 | Neutral |
| 15 | rs375531700 | N105K | 0.4 | Tolerated | 0.386 | Benign | 6 | Neutral |
| 16 | rs375591629 | L132I | 0.08 | Tolerated | 0.706 | Damaging | 6 | Neutral |
| 17 | rs543058073 | A193T | 0.52 | Tolerated | 0 | Benign | 9 | Neutral |
| 18 | rs547943238 | M159L | 0.06 | Tolerated | 0 | Benign | 8 | Neutral |
| 19 | rs548624692 | V22L | 0.63 | Tolerated | 0 | Benign | 5 | Neutral |
| 20 | rs550289178 | L175V | 0.5 | Tolerated | 0.038 | Benign | 9 | Neutral |
| 21 | rs571904031 | A169G | 0.27 | Tolerated | 0.322 | Benign | 7 | Neutral |
| 22 | rs781787508 | A101V | 0.42 | Tolerated | 0.926 | Damaging | 4 | Neutral |
| 23 | rs781794319 | P201L | 0 | Deleterious | 0.642 | Damaging | 3 | Neutral |
| 24 | rs781850242 | N44H | 0.13 | Tolerated | 0.047 | Benign | 8 | Neutral |
| 25 | rs781869508 | K195L | 0.2 | Tolerated | 0 | Benign | 7 | Neutral |
| 26 | rs781894397 | A90S | 0.25 | Tolerated | 0.001 | Benign | 5 | Neutral |
| 27 | rs781898442 | R202L | 0.02 | Deleterious | 0.112 | Benign | 5 | Neutral |
| 28 | rs781904490 | V54M | 0.08 | Tolerated | 0.991 | Damaging | 2 | Neutral |
| 29 | rs781905709 | G177S | 0 | Deleterious | 1 | Damaging | **2** | Disease |
| 30 | rs781937427 | V150M | 0.11 | Tolerated | 0.001 | Benign | 9 | Neutral |
| 31 | rs781950391 | P206L | 0.04 | Deleterious | 0.179 | Benign | 6 | Neutral |
| 32 | rs781958238 | A178V | 0.02 | Deleterious | 0.943 | Damaging | 9 | Neutral |
| 33 | rs781958554 | G59S | 0.09 | Tolerated | 1 | Damaging | 4 | Disease |
| 34 | rs781978821 | P186S | 0.6 | Tolerated | 0.052 | Benign | 7 | Neutral |
| 35 | rs781999702 | L15P | 0 | Deleterious | 0.997 | Damaging | **4** | Disease |
| 36 | rs782012011 | V55A | 0.41 | Tolerated | 0.997 | Damaging | 3 | Neutral |
| 37 | rs782057893 | G19D | 0.02 | Deleterious | 0.54 | Damaging | 4 | Neutral |
| 38 | rs782098175 | I143M | 0 | Deleterious | 0.983 | Damaging | 4 | Neutral |
| 39 | rs782101922 | G100C | 0 | Deleterious | 1 | Damaging | **5** | Disease |
| 40 | rs782104881 | A11V | 1 | Tolerated | 0.008 | Benign | 9 | Neutral |
| 41 | rs782121496 | G205S | 0.36 | Tolerated | 0.052 | Benign | 8 | Neutral |
| 42 | rs782144968 | R202C | 0.02 | Deleterious | 0.999 | Damaging | 5 | Neutral |
| 43 | rs782157164 | N52S | 0.71 | Tolerated | 0.025 | Benign | 6 | Neutral |
| 44 | rs782160881 | A154V | 0.07 | Tolerated | 0.352 | Benign | 6 | Neutral |
| 45 | rs782197903 | S32L | 0.01 | Deleterious | 0.407 | Benign | 2 | Neutral |
| 46 | rs782203771 | V85L | 0.25 | Tolerated | 0 | Benign | 7 | Neutral |
| 47 | rs782208659 | R187H | 0.05 | Deleterious | 0.056 | Benign | 6 | Neutral |
| 48 | rs782211762 | C24G | 0.03 | Deleterious | 1 | Damaging | **7** | Disease |
| 49 | rs782235875 | L129R | 0 | Deleterious | 0.72 | Damaging | **2** | Disease |
| 50 | rs782283901 | P185T | 1 | Tolerated | 0.876 | Damaging | 2 | Neutral |
| 51 | rs782308147 | E188G | 0.3 | Tolerated | 0 | Benign | 8 | Neutral |
| 52 | rs782317608 | A161G | 0.24 | Tolerated | 0.182 | Benign | 7 | Neutral |
| 53 | rs782350219 | Q62R | 0.02 | Deleterious | 0.963 | Damaging | **6** | Disease |
| 54 | rs782368222 | V107E | 0.02 | Deleterious | 0.969 | Damaging | **2** | Disease |
| 55 | rs782379943 | T111M | 0.11 | Tolerated | 0.209 | Benign | 5 | Neutral |
| 56 | rs782383619 | P186Q | 0.6 | Tolerated | 0.458 | Benign | 5 | Neutral |
| 57 | rs782400357 | R157H | 0.08 | Tolerated | 1 | Damaging | 1 | Neutral |
| 58 | rs782433762 | M28T | 0.03 | Deleterious | 1 | Damaging | 3 | Neutral |
| 59 | rs782465492 | T8M | 0.18 | Tolerated | 0.012 | Benign | 7 | Neutral |
| 60 | rs782475802 | A127V | 0.38 | Tolerated | 0.96 | Damaging | 4 | Neutral |
| 61 | rs782486517 | A200V | 1 | Tolerated | 0.046 | Benign | 7 | Neutral |
| 62 | rs782500975 | I45M | 0.48 | Tolerated | 0.0372 | Benign | 8 | Neutral |
| 63 | rs782503893 | S138L | 0.69 | Tolerated | 0.945 | Damaging | 2 | Disease |
| 64 | rs782555330 | A168V | 0.01 | Deleterious | 0.949 | Damaging | 6 | Neutral |
| 65 | rs782583748 | I87V | 1 | Tolerated | 0.631 | Damaging | 9 | Neutral |
| 66 | rs782615892 | A25S | 0.46 | Tolerated | 0.051 | Benign | 7 | Neutral |
| 67 | rs782617402 | V196I | 0.4 | Tolerated | 0 | Benign | 10 | Neutral |
| 68 | rs782651653 | A127S | 0.38 | Tolerated | 0.024 | Benign | 8 | Neutral |
| 69 | rs782652592 | P216S | 0.04 | Deleterious | 0.01 | Benign | 4 | Neutral |
| 70 | rs782657552 | A170V | 0.02 | Deleterious | 0.39 | Benign | 5 | Neutral |
| 71 | rs782664344 | V119E | 0.02 | Deleterious | 0.635 | Damaging | **2** | Disease |
| 72 | rs782752242 | S183L | 0.3 | Tolerated | 0.987 | Damaging | 5 | Neutral |
| 73 | rs782754141 | G117D | 0.02 | Deleterious | 0.489 | Benign | 4 | Disease |
| 74 | rs782810492 | P152T | 0.25 | Tolerated | 0 | Benign | 4 | Neutral |
| 75 | rs782814963 | T10I | 0.51 | Tolerated | 0 | Benign | 2 | Neutral |
| 76 | rs868906959 | R216H | 0.03 | Deleterious | 0.969 | Damaging | 4 | Neutral |
| 77 | rs868962196 | A208S | 0.43 | Tolerated | 0.001 | Benign | 9 | Neutral |
| 78 | rs1003490578 | E153K | 0.91 | Tolerated | 0.115 | Benign | 6 | Neutral |
| 79 | rs1035237771 | I142V | 0.4 | Tolerated | 0.201 | Benign | 9 | Neutral |
| 80 | rs1049954465 | A25V | 0.46 | Tolerated | 0.693 | Damaging | 7 | Neutral |
| 81 | rs1168202234 | G93R | 0.01 | Deleterious | 1 | Damaging | **3** | Disease |
| 82 | rs1174416533 | M3I | 0.4 | Tolerated | 0.001 | Benign | 8 | Neutral |
| 83 | rs1187973447 | V165L | 0.68 | Tolerated | 0 | Benign | 7 | Neutral |
| 84 | rs1199877723 | A128P | 0.01 | Deleterious | 0.763 | Damaging | **3** | Disease |
| 85 | rs1216166758 | L82F | 0.01 | Deleterious | 0.979 | Damaging | 5 | Neutral |
| 86 | rs1219855497 | P149S | 0.09 | Tolerated | 0.684 | Damaging | 5 | Disease |
| 87 | rs1232658070 | Q155L | 1 | Tolerated | 0.01 | Benign | 2 | Neutral |
| 88 | rs1235543238 | D109G | 0.08 | Tolerated | 0.996 | Damaging | 1 | Neutral |
| 89 | rs1243569776 | A78V | 0.01 | Deleterious | 0.946 | Damaging | **1** | Disease |
| 90 | rs1319589309 | A78T | 0.07 | Tolerated | 0.594 | Damaging | 0 | Neutral |
| 91 | rs1340285343 | D145E | 0.22 | Tolerated | 0.006 | Benign | 5 | Neutral |
| 92 | rs1341822731 | V107L | 0.47 | Tolerated | 0.002 | Benign | 7 | Neutral |
| 93 | rs1352314779 | T20M | 0.24 | Tolerated | 0.889 | Damaging | 5 | Neutral |
| 94 | rs1354607793 | K190N | 0.22 | Tolerated | 0 | Benign | 9 | Neutral |
| 95 | rs1360696295 | L180F | 0.17 | Tolerated | 0.883 | Damaging | 4 | Neutral |
| 96 | rs1373740080 | T212S | 1 | Tolerated | 0 | Benign | 9 | Neutral |
| 97 | rs1375975812 | K64E | 0 | Deleterious | 0.996 | Damaging | **5** | Disease |
| 98 | rs1380552982 | G205D | 0.22 | Tolerated | 0.112 | Benign | 6 | Neutral |
| 100 | rs1384865365 | T10A | 0.07 | Tolerated | 0 | Benign | 4 | Neutral |
| 101 | rs1397705319 | R144W | 0 | Deleterious | 0.998 | Damaging | **2** | Disease |
| 102 | rs1399490954 | L94M | 0.35 | Tolerated | 0.797 | Damaging | 8 | Neutral |
| 103 | rs1401007590 | C181W | 0 | Deleterious | 1 | Damaging | **5** | Disease |
| 104 | rs1419376483 | I7M | 0.14 | Tolerated | 0.763 | Damaging | 8 | Neutral |
| 105 | rs1436474524 | S209I | 0.05 | Deleterious | 0.024 | Benign | 6 | Neutral |
| 106 | rs1554626603 | Y219S | 0 | Deleterious | 0.994 | Damaging | **4** | Disease |
| 107 | rs1554626609 | Y214H | 0.07 | Tolerated | 0.988 | Damaging | 2 | Neutral |
| 108 | rs1554626611 | S209C | 0.05 | Deleterious | 0.26 | Benign | 8 | Neutral |
| 109 | rs1554626619 | V196D | 0.22 | Tolerated | 0.07 | Benign | 5 | Neutral |
| 110 | rs1554626624 | K190M | 0.27 | Tolerated | 0.245 | Benign | 9 | Neutral |
| 111 | rs1554626629 | P185H | 0.03 | Deleterious | 0.676 | Damaging | 1 | Neutral |
| 112 | rs1554626645 | A168T | 0.17 | Tolerated | 0.609 | Damaging | 6 | Neutral |
| 113 | rs1554626650 | M159I | 0.06 | Tolerated | 0 | Benign | 6 | Neutral |
| 114 | rs1554626652 | E153G | 0.38 | Tolerated | 0.002 | Benign | 7 | Neutral |
| 115 | rs1554626657 | V151G | 0 | Deleterious | 0.999 | Damaging | **1** | Disease |
| 116 | rs1554626661 | V150A | 0.29 | Tolerated | 0.001 | Benign | 7 | Neutral |
| 117 | rs1554626663 | P149L | 0 | Deleterious | 0.626 | Damaging | **4** | Disease |
| 118 | rs1554626665 | Y147C | 0 | Deleterious | 1 | Damaging | **2** | Disease |
| 119 | rs1554626668 | D145N | 0.01 | Deleterious | 0.023 | Benign | 2 | Neutral |
| 120 | [rs1554626669](https://www.ncbi.nlm.nih.gov/snp/rs1554626669) | R144Q | 0.29 | Tolerated | 0.044 | Benign | 2 | Neutral |
| 121 | rs1554626675 | N140I | 0 | Deleterious | 0.57 | Damaging | **3** | Disease |
| 122 | rs1554626684 | V133L | 0.04 | Deleterious | 0.004 | Benign | 4 | Neutral |
| 123 | rs1554626686 | T131I | 0.26 | Tolerated | 0.002 | Benign | 3 | Neutral |
| 124 | rs1554626694 | T117N | 0.03 | Deleterious | 0.353 | Benign | 0 | Disease |
| 125 | rs1554626695 | K115N | 0.06 | Tolerated | 0.999 | Damaging | 0 | Disease |
| 126 | rs1554626702 | N105S | 0.4 | Tolerated | 0.837 | Damaging | 7 | Neutral |
| 127 | rs1554626707 | A101S | 0.42 | Tolerated | 0.01 | Benign | 6 | Neutral |
| 128 | rs1554626722 | A91S | 0.02 | Deleterious | 0.003 | Benign | 8 | Neutral |
| 129 | rs1554626727 | A79T | 0.24 | Tolerated | 0.912 | Damaging | 7 | Neutral |
| 130 | rs1554626735 | S68L | 0 | Deleterious | 1 | Damaging | **5** | Disease |
| 131 | rs1554626749 | A33T | 0.01 | Deleterious | 0.962 | Damaging | **2** | Disease |
| 132 | rs1554626750 | P27R | 0 | Deleterious | 0.984 | Damaging | **6** | Disease |
| 133 | rs1554626752 | P27S | 0 | Deleterious | 1 | Damaging | **4** | Disease |
| 134 | rs1554626760 | L18M | 0.03 | Deleterious | 0.912 | Damaging | 7 | Neutral |
| 135 | rs1554626763 | W17C | 0 | Deleterious | 1 | Damaging | **4** | Disease |
| 136 | rs1554626768 | V14L | 1 | Tolerated | 0.001 | Benign | 6 | Neutral |
| 137 | rs1554626776 | L5V | 0.01 | Deleterious | 0.18 | Benign | 9 | Neutral |
| 138 | rs1554626778 | S2P | 0.01 | Deleterious | 1 | Damaging | 0 | Neutral |
| 139 | rs1584302554 | N148T | 0.01 | Deleterious | 0.944 | Damaging | 3 | Neutral |
| 140 | rs1584302838 | E47D | 0.05 | Deleterious | 0.364 | Benign | 3 | Neutral |

Suppl. Table 3: Quality metrics of homology modeling parameters.

| Model Name | GMQE Score | QMEAN4 Score | Seq. Identity | Coverage |
| --- | --- | --- | --- | --- |
| CLDN-3 | 0.67 | ~0.66 | 92.35% | 0.83 |
| C181W | 0.66 | ~0.69 | 92.35 | 0.83 |
| A128P | 0.66 | ~0.68 | 91.80% | 0.83 |
| A78V | 0.67 | ~0.68 | 91.80% | 0.83 |
| N140I | 0.67 | ~0.69 | 91.80% | 0.83 |
| Y147C | 0.67 | ~0.68 | 91.80 | 0.83 |
| G100C | 0.66 | ~0.68 | 91.80 | 0.83 |

Suppl. Table 4: Total H-bond formation for CLDN-3 along with its nsSNPs during the MDS analysis

| **S.No.** | **nsSNP ID** | **Amino acid mutation** | **Total H-bond** |
| --- | --- | --- | --- |
| 1 | rs1401007590 | C181W | 2687 |
| 2 | rs1199877723 | A128P | 2640 |
| 3 | rs1243569776 | A78V | 2771 |
| 4 | rs1554626675 | N140I | 2817 |
| 5 | rs1554626665 | Y147C | 2725 |
| 6 | rs782101922 | G100C | 2811 |

** CLDN-3 Total H-Bonds: 2645
